# Professor Tom Connors and the development of novel cancer therapies by the Phase I/II Clinical Trials Committee of Cancer Research UK

**DOI:** 10.1038/sj.bjc.6601106

**Published:** 2003-07-29

**Authors:** D R Newell, K M Searle, N B Westwood, S S Burtles

**Affiliations:** 1 Northern Institute for Cancer Research, University of Newcastle upon Tyne, Framlington Place, Newcastle upon Tyne NE2 4HH, UK; 2 Drug Development Office, Cancer Research UK, PO Box 123, 61 Lincoln's Inn Fields, London WC2A 3PX, UK

On the 7th of May 2002 a conference of the Cancer Research UK Phase I/II Clinical Trials Committee was held to commemorate the achievements of the late Professor Tom Connors in the field of cancer drug development. Tom Connors died on the 4th of February 2002 from the disease he had spent his life studying, and obituaries chronicling his many scientific and personal attributes and contributions have been written (Double, 2002; Newell, 2002).

Arguably Tom's greatest achievement was the establishment of the Cancer Research UK, formerly Cancer Research Campaign (CRC), Phase I/II Committee with Laszlo Lajtha and Brian Fox in 1980. Hence, the Committee Tom had created and steered to outstanding international recognition and scientific success dedicated the first meeting after his death to reviewing the laboratory and clinical research that Tom had undertaken and made possible. The meeting was both a retrospective review and a forward look at developmental therapeutics in all its forms: small molecule, macromolecule, gene therapy and immunological. Peppered throughout the presentations were not only references to Tom's scientific contributions, but also anecdotes and reminiscences collected during 30 years of cancer research, these brought both smiles and tears of laughter to the assembled committee members, and to his widow, Pearl, and daughter, Clare, who were there.

As Gordon McVie, the former Director General of the Cancer Research Campaign, who strongly supported the Committee's activities, pointed out in his summary, there can be very few who have contributed as much as Tom Connors to cancer research. This article reflects those aspects of Tom's work related to the activities of the Cancer Research UK Phase I/II Clinical Trials Committee.

## **TOM CONNORS AND THE CANCER RESEARCH CAMPAIGN, NATIONAL CANCER INSTITUTE USA AND EUROPEAN ORGANISATION FOR RESEARCH AND TREATMENT OF CANCER**–Trevor Hince, Omar Yoder and Herbie Newell

Tom Connors was an advisor, supporter and friend of the Cancer Research Campaign (CRC) for over 30 years serving as a member and/or Chairman of the Scientific, Phase I/II Clinical Trials and Gibb Fellowship Committees. In recognition of his life-time contribution he was made an Emeritus Fellow of the CRC in 1998. The first meeting of the Phase I/II Committee was held in July 1980. The second meeting in October 1981 set itself the challenge of selecting no fewer than 20 compounds for clinical trials in 2 years with what is, by current standards, a very modest funding of £41 000 (∼$ or Euro 60 000) *per annum*. At the outset, the Committee identified four key needs that were major barriers to academic cancer drug development:

- To stimulate the submission of compounds for Phase I testing.
- To simplify and provide access to preclinical toxicology.
- To develop clinically tractable formulations for new drugs that complied with regulatory requirements.
- To open a dialogue with the Committee on Safety of Medicines in order to establish a legal framework within which clinical trials with academic drugs could be undertaken.

By December 1983 the Phase I/II Committee was able to report to the CRC Scientific Committee that two compounds had entered clinical trials and nine were undergoing preclinical development, and a Clinical Data Centre directed by Edward Newlands had been established at the Charing Cross Hospital. In the intervening 20 years, the Committee has selected 89 agents for clinical trials: 25 cytotoxic agents, five antiendocrine drugs, 28 small molecules with novel or unknown mechanisms of action, five polymer-targeted agents and 26 antibody-targeted agents/immunotherapies. To date, four agents have progressed to the market with two, temozolomide (**I**–Temodal®) and 4-hydroxyandrostenedione (**II**–Lentaron®), being particularly successful and many more showing promising activity in Phase I, II and III trials.


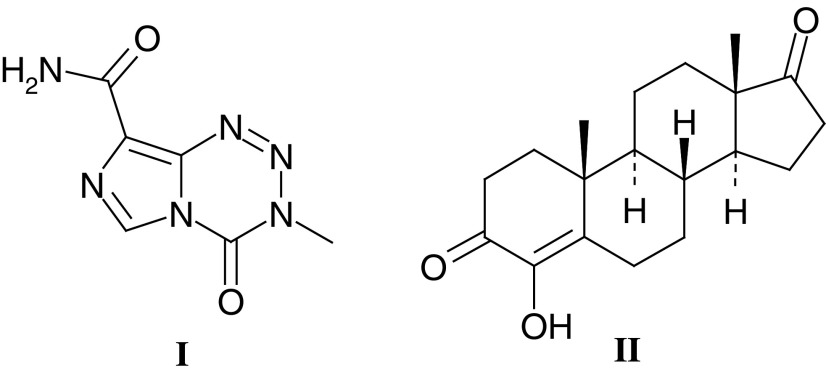


Although Tom never worked outside the UK, he was in every sense a truly international scientist. In particular, his long association with the National Cancer Institute, US (NCI) began in 1966 at the Ninth International Cancer Congress in Tokyo when he met John Venditti, who was then chief of drug evaluation at the NCI. In the years that followed Tom became a key adviser to the NCI's developmental therapeutics and toxicology programmes. He paved the way for the NCI's highly successful international alliances, and initiated the collaboration with the CRC.

Tom had the distinction of being the first ‘international figure’ to serve on the NCI's top scientific advisory and oversight committees. He also freely offered his wise counsel and expert knowledge to the NCI in-house panels, programme directors and countless collaborators and colleagues. It was through his efforts that the NCI was able to convince the FDA to take a more open view on approving IND applications for anticancer drugs from expert groups in Europe and the UK.

When the NCI launched its European Programme and opened the Brussels Office in 1972, Tom became the principal adviser in planning its long-term strategy. He fostered a highly successful transatlantic collaboration and helped the NCI bridge the gap with Eastern Europe, and establish links with the European Organisation for Research and Treatment of Cancer (EORTC). Through his efforts, the NCI was by now well represented in the EORTC drug development groups and its programme for developing new European anticancer agents rapidly gained momentum. Given this flow of new compounds from European sources to the NCI, on Tom's advice, the NCI established a testing laboratory at the Institut Jules Bordet in Brussels to screen new agents. By the late 1970s, tens of thousands of new molecules had been tested by NCI Brussels and new agents active in the screens were selected for clinical trials.

Although anticancer drug development was well underway in Europe, the scientists with expertise in experimental therapeutics were predominantly from one country, the UK. Therefore, Tom saw the opportunity to establish a drug development network in the UK and presented his plan to the NCI. It was viewed with favour and NCI compounds were selected for consideration by the CRC Phase I/II Committee. Until that time it had been inconceivable for the NCI to give new active compounds from its screens to ‘offshore developers’ and no other individual deserves more credit in setting the stage for the NCI's early international success in anticancer drug research. He was a facilitator, a trusted colleague and collaborator, inspiring all who surrounded him. He lived the working philosophy that he taught: ‘Science should be both satisfying and fun; any new findings must be shared, openly exchanged, and credit given where due.’

Tom was also an active member of the EORTC for many years, being elected Chairman of the Laboratory Research Division in 1993, as well as the EORTC Board from 1991 to 1996. Tom saw the success of the CRC Phase I/II Clinical Trials Committee as a model on which to build and encouraged close collaboration, under the umbrella of the tripartite CRC/NCI/EORTC agreement, with European drug developers across the continent.

## CANCER DRUG DEVELOPMENT IN THE UK IN THE ACADEMIC SECTOR

**The Cancer Research UK Drug Development Office**–David Secher and Sally Burtles

The Drug Development Office (DDO) was set up in 1992 in order to introduce internationally accepted quality standards into Phase I/II Committee trials to ensure that the data produced by the Committee would be acceptable to the pharmaceutical industry. It was anticipated that, if Phase I/II Committee trials were carried out to Good Clinical Practice (GCP) standards, then time, money and, ultimately, patients' lives could be saved, and Tom Connors was pivotal in having this vision.

To illustrate the importance of performing studies to appropriate standards, it is instructive to consider the case of temozolomide. As described below, temozolomide was discovered by Malcolm Stevens and colleagues at Aston University. The drug was taken through preclinical development and then tested in Phase I and II clinical trials by the Committee. In 1992, the Phase I/II Committee completed the Phase I trial of temozolomide that established the maximum tolerated dose (MTD), its toxicity profile and a recommended dose for Phase II studies (Newlands *et al*, 1992). After demonstrating the utility of temozolomide in patients with glioma, the drug was licensed to Schering-Plough, who felt it necessary to repeat the studies performed by the Phase I/II Committee before embarking on pivotal Phase III trials (Dhodapkar *et al*, 1997). These studies identified the same MTD, toxicity profile and recommended dose. Had the Phase I/II Committee trials been done to GCP, temozolomide, now Temodal®, could have been launched 2 years earlier, and more patients could have benefited from its treatment.

Learning from this experience, and recognising the need to undertake clinical studies to contemporary regulatory standards, the DDO was set up to implement systems to ensure that trials are carried out to ICH-GCP (International Committee on Harmonisation of GCP) standards. In 2000, the DDO volunteered for a GCP inspection by the Medicines Control Agency and, while identifying areas for improvement, the inspection was a resounding success.

In 2001, the European Union (EU) published a Directive on clinical trials that will be implemented by May 2004. The key issues for Cancer Research UK in complying with the European Directive (European Parliament, 2001) are that all trials are to be undertaken to GCP, all products are to be made to Good Manufacturing Practice (GMP) standards in a licensed facility, and the option of performing clinical trials in the UK under a Doctors and Dentists Exemption (DDX) will no longer be available.

The GCP requirement has enormous implications for academic clinical research in general. However, it is likely to be less of an issue for Phase I/II Committee trials as the DDO already largely operates to these standards. GCP standards also cover laboratory studies undertaken as part of clinical trials and, in anticipation of this, Cancer Research UK has employed a Quality Assurance Manager to ensure that laboratory data are also generated to the required standards.

In addition to providing access to preclinical toxicology (see below) and high-quality clinical trials management, Cancer Research UK can produce both small molecule products and biological agents to GMP or equivalent standards. The Cancer Research UK Formulation Unit at the University of Strathclyde is already licensed to manufacture clinical trials material, and the Biotherapeutics Development Unit at the Cancer Research UK London Research Institute (Clare Hall) will also be licensed before the EU legislation is implemented.

The loss of the DDX is potentially the biggest threat to academic drug development and early clinical trials in the UK. The details of the requirements for the new legislation are still unknown, but will almost certainly entail more extensive preclinical testing than currently undertaken by Cancer Research UK. While any additional work can be performed, it may increase the costs significantly and delay the clinical evaluation of new treatments. The additional studies are also likely to involve more experiments in animals, and may require studies in non-rodent species. As discussed below, the Phase I/II Committee has pioneered the use of rodent-only toxicology, an issue that Tom felt passionate about, and any move towards the use of more laboratory animals, particularly non-rodents, should only be countenanced if there is an unequivocal scientific rationale.

Recent advances in molecular genetics, and the improved understanding of the pathology of cancer that these advances have allowed, are already identifying new classes of agents for cancer treatment. Since these therapies are designed to be selective for specific molecular pathologies they will need to be evaluated in genetically defined subsets of patients. As discussed below, the development of ‘targeted therapies’ will throw up many new challenges for the drug development community. Thanks to Tom's vision, the Cancer Research UK DDO is well placed to respond and continue to be a leader in anticancer drug development in this new millennium.

***In vivo* tumour models and preclinical toxicology studies**–John Double and Herbie Newell

For a candidate drug to be considered for clinical evaluation, it is mandatory that there is a reasonable expectation that biological activity will be seen in patients at tolerated doses. *In vivo* tumour models are used to demonstrate that activity can be achieved in rodents, normally mice, bearing either rodent or human tumours. In the era of cytotoxic drug development, tumour growth inhibition and regression was the biological end point most frequently used in preclinical studies. However, with the advent of targeted therapies, mechanistic studies have replaced tumour growth inhibition as the primary *in vivo* preclinical end point. Thus the initial objective of mechanistic *in vivo* studies is to show that a drug can interact with its intended target at tolerated doses. If target interaction can be shown, a link to the desired biological effect of the agent is sought, for example inhibition of tumour growth, invasion, angiogenesis or metastasis for which orthotopic models are now widely used. In addition, the availability of mice with targeted gene disruption (knockout mice) or gene insertion (knockin mice), as well as the use of tumours with defined molecular genetics, means that host–tumour interaction can be studied in robust and predictive models.

The introduction of high-throughput screening has also been an important development in cancer drug discovery, and the NCI *in vitro* cell line panel has become a particularly valuable resource (Monks *et al*, 1997). However, the compounds identified still have to demonstrate activity in an appropriate *in vivo* model. Because of the large number of compounds that can be identified by *in vitro* screening strategies, the use of conventional tumour models is not appropriate, since large numbers of animals would be required. To address this problem, the *in vivo* hollow fibre model has been developed and this can complement mechanism-based *in vivo* models in preclinical drug development (Hollingshead *et al*, 1995; Suggit *et al*, 2002).

The preclinical data package for therapies considered by the Cancer Research UK Phase I/II Committee, and more recently the New Agents Committee, requires:

- A description of the drug target and how it has been validated.
- Information on the mechanism of action of the new agent.
- Evidence that the drug has activity in an *in vivo* model at tolerated doses and details of the pharmacokinetics of the agent at active doses.
- Experimental data to show that biological activity is linked to the proposed mechanism of action.
- Details, preferably supported by validation data, of how the pharmacokinetics and pharmacodynamics or immunodynamics of the agent will be studied in the clinical trial.

If these data are felt to be robust, the agent is selected for clinical trials prior to which key preclinical development steps are undertaken. The resources to complete these preclinical steps, notably toxicology and bulk manufacture/formulation, were provided for the first time in a noncommercial setting in the UK by the Phase I/II Committee in 1980. Toxicology in particular can be a contentious issue, and at the time the Committee was established, lengthy and expensive protocols, often involving large numbers of animals, rodent, non-rodent and even primates, were standard practice. With Brian Fox, Tom developed simplified rodent-only toxicology protocols (EORTC/CRC, 1990) and these were used until 1995. A recent review of the data on the first 25 compounds to be studied (Newell *et al*, 1999) confirmed that rodent-only toxicology provides a safe Phase I trial starting dose and predicts toxicity for the vast majority of the agents investigated. These data were considered by the European Medicines Evaluation Agency Committee on Proprietary Medicinal Products (EMEA CPMP) Safety Working Party, and contributed to the guidelines that include the use of rodent-only toxicology studies for first-in-human trials with direct acting anticancer agents (European Agency for the Evaluation of Medicinal Products, 1999). Subsequent toxicology studies performed by the Phase I/II Committee have focussed on the development of compound-orientated protocols in which the intended clinical route and schedule of administration is mirrored as closely as possible in the preclinical safety studies (Burtles *et al*, 1995). This approach, coupled with innovative clinical trial designs that promote patient enrolment to doses that are likely to be effective, and the use of clinical centres with proven expertise in early clinical trials, ensures that the need to select a safe starting dose and animal welfare issues are appropriately balanced.

**Clinical trial design and end points**–John Smyth, Ian Judson, Duncan Jodrell and Pat Price

Early clinical trial design has developed dramatically over the past 20 years, and in particular, end points in Phase I trials have changed from toxicity to pharmacodynamics. Although safety remains paramount, the recognition that response does not relate directly to toxicity even with conventional cytotoxic drugs such as carboplatin (Jodrell, 1999), resulted in a reappraisal of the objectives and design of early clinical trials. Apart from the simplistic appeal of end points other than toxicity (trials ‘designed’ to make patients sick are unacceptable ethically), targeted therapies are in many cases predicted to require long-term administration so that traditional Phase I trial end points of MTD and dose-limiting toxicity have been replaced by the optimal biological dose and pharmacodynamics/immunodynamics. The optimal biological dose can be defined as that which results in drug levels in the blood and/or tumour, which produced activity in preclinical models, the maximal change in the level of a surrogate marker or, if it can be measured directly, the desired clinical effect. Once the optimal biological dose has been identified, Phase II trials can be performed and historically these have used response rates as a surrogate marker for patient survival. A key recent additional component of Phase II trials is to define the presence of the drug target in the tumour and to relate target levels to clinical activity. Such analyses have been pivotal to the development and subsequent clinical use of drugs such as trastuzumab and imatinib, and are now included wherever technically feasible in both Phase I and Phase II trials.

In addition to providing proof of principle at an early stage of the clinical evaluation of a compound, knowledge of the time course and dose dependency of pharmacodynamic effects can help to optimise scheduling. A good example comes from the Phase I/II Committee trial of the thymidylate synthase (TS) inhibitor nolatrexed (**III**–AG337, Thymitaq) where the rapid recovery of plasma deoxyuridine levels (a surrogate marker of systemic TS inhibition) at the end of a 24 h infusion led to the investigation of 5 day i.v./p.o. and 10 day p.o. regimens (Rafi *et al*, 1995, 1998; Jodrell *et al*, 1999; Hughes *et al*, 2000). The 5 day i.v. regimen was ultimately selected for Phase II and Phase III trials.

With targeted agents the challenge is to design and validate pharmacodynamic markers, and these tend to be compound specific and often require lateral thinking in order to provide a clinically feasible assay. An excellent example from the work of the Phase I/II Committee relates to the Phase I trial of an antagonist of mitogenic neuropeptide growth factors, substance P antagonist G (**IV**–SPAG, Cummings *et al*, 1999; Clive *et al*, 2001). In this study, forearm blood flow/venous plethysmography was used to show that SPAG levels could be achieved in patients, which blocked substance P-induced vasodilation.

Pharmacodynamic studies are now a mandatory component of early clinical trials and, while surrogate markers such as those used for nolatrexed and SPAG are valuable, direct measures of drug action in the tumour are preferable. As a consequence, noninvasive techniques such as magnetic resonance imaging and spectroscopy (MRI/MRS) and positron emission tomography (PET) have been developed and are now increasingly used in early clinical trials (see below). Where it is not possible to develop a noninvasive approach to pharmacodynamic monitoring, a surgical biopsy can be required. Invasive surgical procedures require special justification, and this includes robust preclinical assay validation and, if possible, demonstration that the pharmacodynamic effect can be observed in peripheral blood cells in patients at tolerated doses. Recent Phase I/II Committee studies with 17-allylamino, 17-demethoxy geldanamycin (17-AAG) (**V**), an antagonist of the HSP90 molecular chaperone important in the stabilisation of a number of oncogenic proteins, has involved surgical biopsy and the analysis of HSP90 client protein levels following drug treatment (Banerji *et al*, 2002). At a dose of 320 mg m^–2^ 17-AAG clear effects on Raf-1, HSP70 and CDK4 levels have been seen, and these studies provide an excellent example of contemporary Phase I trial design.


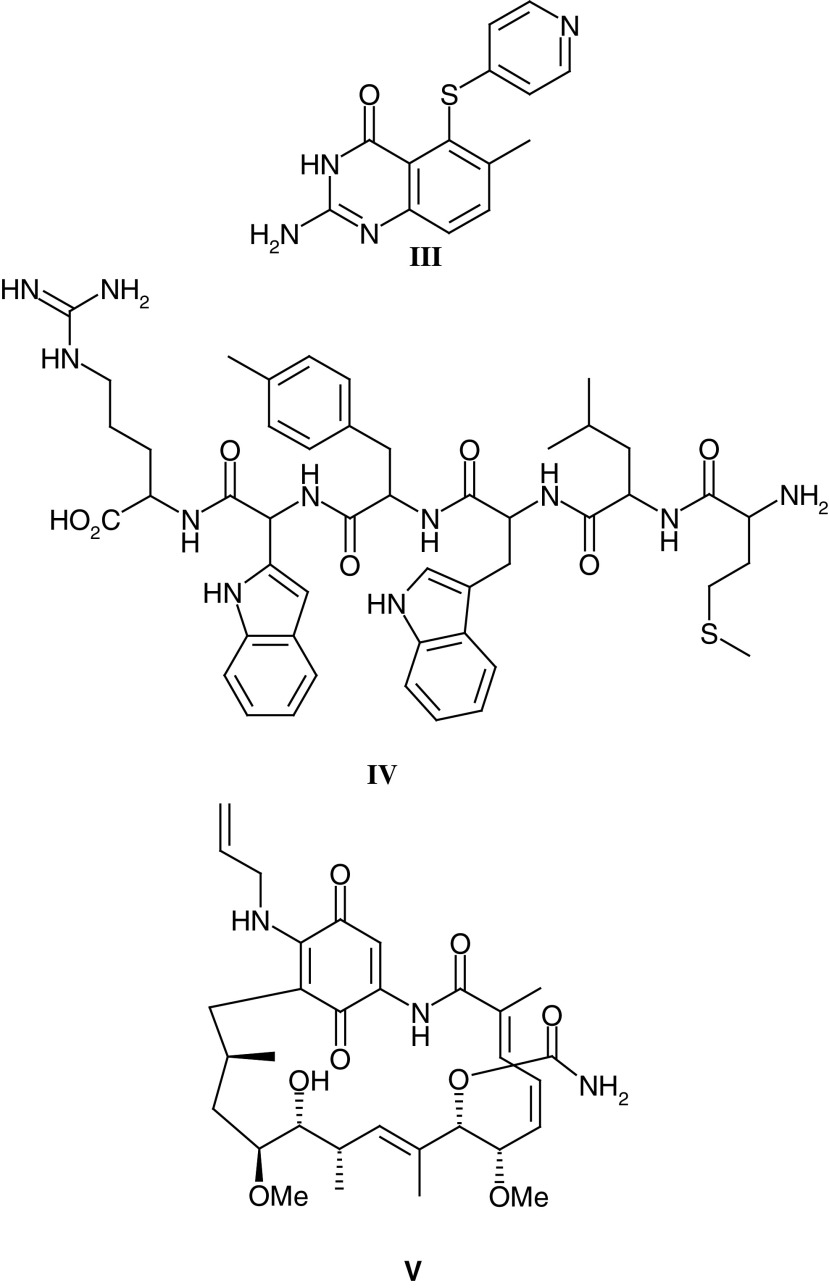


Noninvasive techniques are always preferable to surgery, and the potential of functional imaging using PET was recognised by Tom Connors in the late 1980s. In 1991, the CRC supported Pat Price and Terry Jones, at the MRC Cyclotron Unit, Hammersmith Hospital, to undertake the synthesis of positron emitting forms of novel antitumour agents undergoing evaluation by the Phase I/II Committee. The successful synthesis of both ^11^C-DACA (**VI**) and ^11^C-temozolomide (**I**) led to a number of ‘world-first’ noninvasive pharmacokinetic and pharmacodynamic studies. Parallel studies with ^18^F-fluorodeoxyglucose as an early marker of tumour response and ^11^C-thymidine as a measure of TS inhibition following nolatrexed treatment confirmed the potential of PET as a noninvasive pharmacokinetic/dynamic tool. Most recently, the Phase I/II Committee has studied the antivascular agents DMXAA (**VII**) and combretastatin A4 phosphate (**VIII**), and measurements of tumour blood flow by PET as well as MRI (see below) have been critical in defining the clinical pharmacodynamics of these antivascular agents.


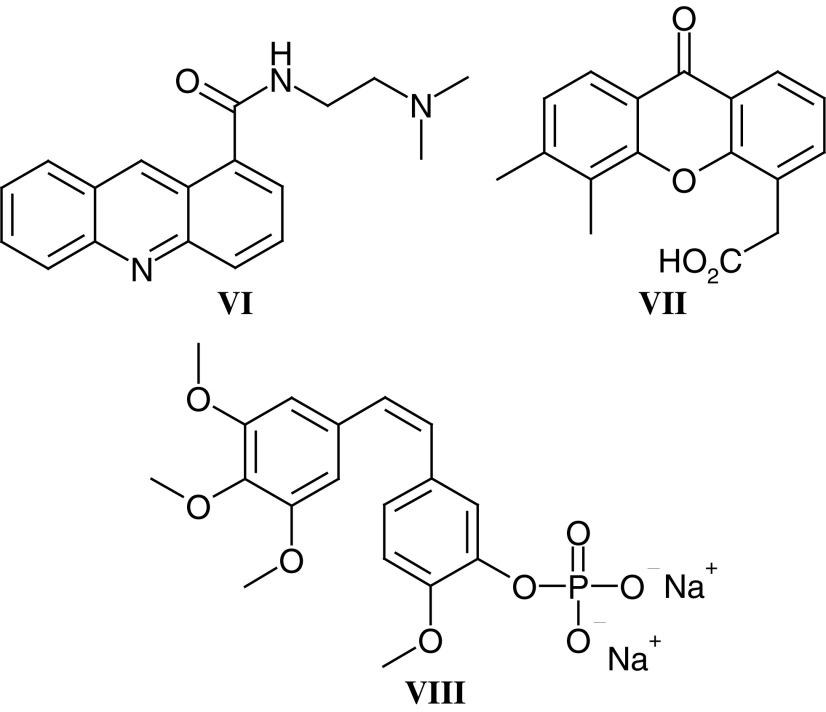


## THE EVALUATION OF APPROACHES DESIGNED TO ACHIEVE TUMOUR SELECTIVE DRUG ACTIVATION AND DELIVERY

**Tumour selective drug activation: from aniline mustard to state-of-the-art bioreductive agents**–Paul Workman and Ian Stratford

One of Tom Connor's own spectacular successes in elucidating the mechanism of tumour-specific selectivity involved the overexpression of a prodrug activating enzyme in certain cancers. In 1965, Tom and Max Whisson reported the regression and cure of established ADJ-PC5 mouse plasma cell tumours by a single dose of aniline mustard (**IX**; Connors and Whisson, 1965a, 1965b). They showed that the mechanism underlying the extraordinary selectivity of aniline mustard was its metabolism by hydroxylation and glucuronide conjugation in the liver, followed by hydrolysis to the highly reactive and potent metabolite *p*-hydroxylaniline mustard in tumours expressing high levels of *β*-glucuronidase (Connors and Whisson, 1966; Connors *et al*, 1973). On the basis of this work, aniline mustard progressed to clinical trials. The work also led to the synthesis of the glucuronide, sulphate and phosphate derivatives of *p*-hydroxyaniline mustard as prodrugs for tumour-selective activation in cancers rich in glucuronidase, sulphatase and phosphatases, respectively (Double and Workman, 1977). One of the limitations of the approach was the potential for cleavage of the prodrugs in normal tissues and plasma, as well as for irreversible inhibition of the activating enzyme (Workman, 1978; Workman and Double, 1978). Nevertheless, Tom's work on these and other tumour-activated prodrugs laid the foundations for the development of antibody directed enzyme prodrug therapy (ADEPT) and gene directed pro-drug therapy (GDEPT) (see sections below), and his studies directly relate in concept to the development of bioreductive prodrugs to exploit tumour hypoxia and the overexpression of certain reductase enzymes in a high proportion of cancers.


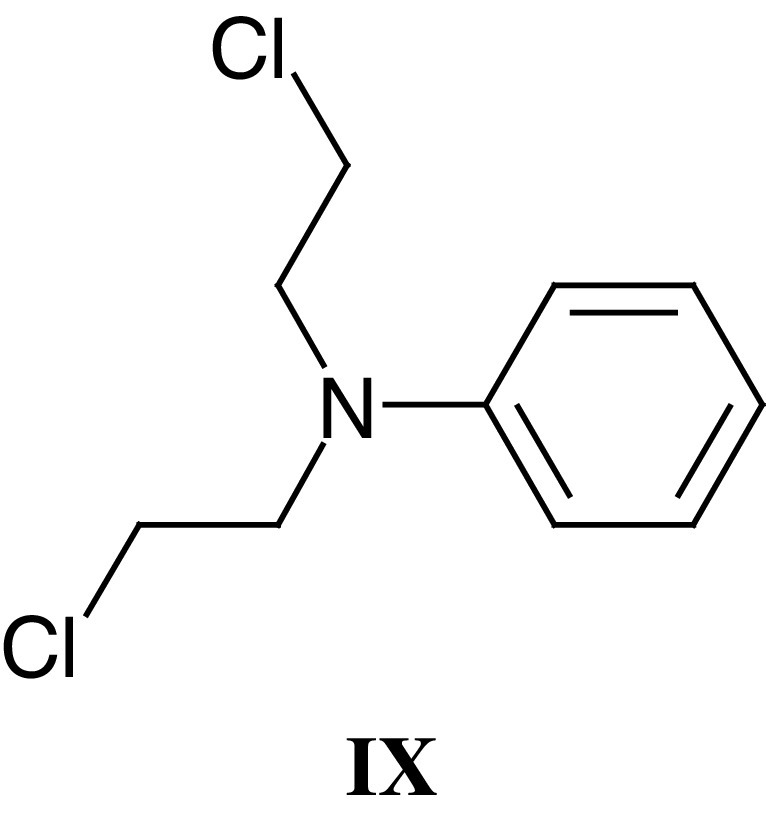


Following on from work pioneered by the late Ged Adams, many attempts have been made to overcome and exploit tumour hypoxia both because it is a unique feature of tumours, as opposed to normal tissues, and because hypoxia underlies the radio-resistance of many solid tumours. The most widely studied class of hypoxic cell sensitisers, that is agents designed to increase the activity of radiation and/or drug therapy in hypoxic cells, are the nitroimidazoles. With nitroimidazoles, definition of the relationship between one-electron reduction potential and the concentration required for a given degree of enhancement identified optimal physicochemical characteristics for radiation sensitisation (Adams *et al*, 1977). Furthermore, these studies with radiosensitisers led to the development of bioreductive hypoxic cell cytotoxins with inherent activity against hypoxic cells (Stratford and Workman, 1998). The aziridinydinitrobenzene derivative CB1954 (see also below and **XV**) and the alkylating nitroimidazole RSU1069 (**X**) were two compounds identified in early studies, and RSU1069 was selected for clinical trials by the Phase I/II Committee in 1982, and completed in 1986. Unmanageable nausea and vomiting was encountered with RSU1069 (Horwich *et al*, 1986); however, the bioreductive cytotoxins tirapazamine and AQ4N (**XI**) (Patterson and McKeown, 2000), the latter currently in clinical trials through the Phase I/II Committee, testify to the continued interest in the area of hypoxia-directed therapy. Tirapazamine has reached Phase III trials where initial results in combination with cisplatin have been encouraging.

A more sophisticated approach to exploiting tumour hypoxia is the combination of a therapeutic entity, either a cytotoxin or a more subtle agent designed to exploit tumour biology, with an inactivating bio-reductive trigger. Reduction of the trigger releases the therapeutic entity and studies with mustard-based systems where the trigger is ‘activated’ by one-electron reduction have resulted in nitrogen : air sensitisation ratios of 485, *vs* 1.5 for the active mustard (McNally *et al*, 2002).

In the development of hypoxia-targeted therapies, an ability to measure tumour oxygen tension in clinical trials is essential, in order to explore relationships between any efficacy observed and the degree of tumour hypoxia. Both invasive studies on tumour biopsies (ie immunohistochemical detection of nitroimidazole binding, eg with pimonidazole and EF5) and noninvasive methods (ie MRS or PET with fluorinated nitroimidazoles such as SR4554 (**XII**) have been evaluated. SR4554, has shown considerable promise in animal models (Aboagye *et al*, 1997, 1998 Seddon *et al*, 2002a) and is currently the subject of a Phase I/II Committee Phase I trial in which MRS is used in patients to detect SR4554-derived signal, indicative of tumour hypoxia (Seddon *et al*, 2002b).


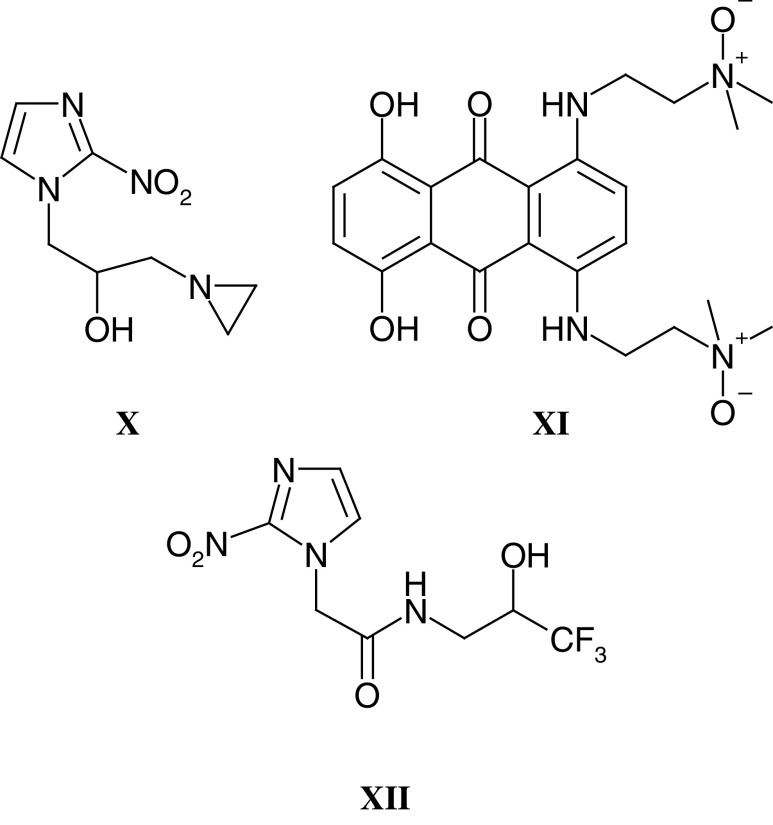


Tumour hypoxia is now well established as a clinical phenomenon, and recent studies have confirmed that the biochemical as well as physiological consequences of hypoxia offer potential for exploitation in prognosis. For example, demonstration that pimonidazole staining and expression of the glut-1 glucose transporter, coupled to the strong relationship between metastasis-free survival in advanced cervical carcinoma and glut-1 expression (Airley *et al*, 2001), suggest a mechanistic rationale for the noninvasive measurement of glut-1 levels by ^18^F-fluorodeoxyglucose PET scanning.

The development of bioreductive agents as tumour-selective prodrugs is not solely restricted to the exploitation of tumour hypoxia. Cancer cells frequently overexpress reductive enzymes, particularly the quinone reductase DT-diaphorase or NQO1 (Fitzsimmons *et al*, 1996). This led to the concept of enzyme-directed bioreductive drug development (Workman, 1994). A number of agents undergo activation by NQO1, including the clinically used agent mitomycin C, simpler quinones such as AZQ, RH1 and EO9, and also CB1954 (Riley and Workman, 1992; Fitzsimmons *et al*, 1996; Sharp *et al*, 2000, see below). EO9 was developed for clinical trials under the auspices of EORTC (Hendriks *et al*, 1993; Plumb and Workman, 1994). Clinical results were disappointing, probably because of the rapid clearance of the drug (Workman *et al*, 1992), but the agent may find a role in the treatment of DT-diaphorase-rich bladder cancers by intravesicular administration.

Intriguingly, DT-diaphorase expression was also found to sensitise cancer cells to the benzoquinone ansamycin 17-AAG (**V**; Kelland *et al*, 1999), which has now entered clinical trial as the first-in-class inhibitor of the HSP90 molecular chaperone ATPase activity (see earlier section on clinical trial design and end points). The mechanism behind this potentiation by DT-diaphorase may relate to reduction of the benzoquinone, but the mode of action of 17-AAG in DT-diaphorase-rich cells remains HSP90 inhibition, leading to depletion of oncogenic client proteins and simultaneous inhibition of the PI3 kinase and RAS-ERK signal transduction pathways (Kelland *et al*, 1999; Clarke *et al*, 2000; Hostein *et al*, 2001). Tom Connors was initially sceptical about our ability to fully understand the mechanism of the antitumour action of even the most exquisitely designed agent (see The Connor's Rules of Anticancer Drug Development, Table 1

**Table 1:** The Connors rules of anticancer drug development

1.	Scientists under the age of 40 believe that cancer research began around 1975
	• It was around 1975 that computerised data searches became generally available
2.	Anticancer agents based on an elegant working hypothesis are sometimes successful in the clinic
	• However, the way they act is often nothing to do with the working hypothesis
3.	There is no direct correlation between the elegance of a working hypothesis and success in the clinic
	• Some cynics believe the correlation is inverse
4.	Results obtained using isolated systems, for example receptors, do not extrapolate readily to cells in culture
	• Results obtained using cells in culture do not extrapolate readily to *in vivo* systems, for example rodents with tumours
	• Results using rodents with tumours do not extrapolate readily to the clinical situation
5.	State of the art technology is vitally important for cancer research
	• State of the art technology complements rather than substitutes for innovative thinking
6.	How to keep happy (adapted from an old Chinese proverb)
	• For one day, get drunk
	• For one week, kill a pig
	• For one month, get married
	• For a lifetime, do cancer research

Personal communication from Professor Tom Connors presented in his George and Christine Sosnovsky Award Lecture, Forty Years of Cancer Research (1999).

). However, he showed a keen interest in the new generation of agents, such as 17-AAG and also imatinib, trastuzumab and Iressa, which are being developed to exploit the specific molecular pathology of cancer as the basis of their therapeutic selectivity, and he was delighted to see the success of this new approach (Workman and Kaye, 2002).

**Antibody enzyme prodrug therapy**–Caroline Springer andRichard Begent

Tom Connors saw the potential of selective prodrug activation in the tumour at a very early stage in his career and was publishing on the subject in 1969. His interests included nitroreductase and microsomal activation of cyclophosphamide; however, targeting an enzyme to the tumour did not become feasible until the 1980s when Ken Bagshawe's CRC laboratories at Charing Cross Hospital described antibody-directed enzyme prodrug therapy (ADEPT) (Bagshawe, 1987; Bagshawe *et al*, 1988). The Phase I/II Committee chaired by Tom, encouraged clinical trials of ADEPT on the mechanism of action of the components to show whether conditions for effective therapy were being achieved.

The ADEPT approach separates the cytotoxic prodrug from the targeting antibody in a two-phase system that has benefits over a one phase chemoimmunoconjugate, immunotoxin or radioimmunoconjugate. The antibody binds to an antigen that is preferentially expressed on the surface or in the interstitial spaces of the tumour cells. The antibody–enzyme complex is administered and allowed to accumulate at the tumour site. Time is allowed for the clearance of the conjugate from blood and normal tissues. Then, a latent nontoxic prodrug is administered, which is converted selectively by the enzyme at the tumour site into a low molecular weight toxic drug (Bagshawe, 1987; Bagshawe *et al*, 1988). The amplification feature inherent in the enzyme of the conjugate molecule system means that one molecule of enzyme can catalyse the conversion of many molecules of the prodrug into the cytotoxic drug, and thereby provides major advantages. Furthermore there is a bystander effect, whereby those cells that are not targeted by the antibody–enzyme conjugate are killed by drug released from neighbouring cells that have been targeted.

The ADEPT approach was initiated using alkylating agent-derived prodrugs (Bagshawe *et al*, 1988 1991; Mann *et al*, 1990; Springer *et al*, 1990) and carboxypeptidase G2 (CPG2) as the activating enzyme (Melton *et al*, 1990). The rationale for the use of alkylating agents as drugs in this approach was that:

- The active drugs show cell-cycle-independent activity.
- The drugs exhibit a dose-dependent effect and are active against quiescent cells.
- The active drugs induce less acquired resistance than other classes of chemotherapeutic agents.
- Chemical deactivation is possible by functional groups that are cleavable by appropriate conjugated enzymes.
- Differences of greater than 100-fold in chemical reactivity between the prodrug and active drug can be achieved.
- Active drugs with very short half-lives can be designed.

In order to derivatise the nitrogen mustard drugs into prodrugs for activation by CPG2, an amidic cleavable bond was introduced between the mustard aromatic ring and a glutamic acid moiety. An advantage of this system was that the prodrug uptake into cells (ie penetration across cell membranes) could be minimised due to the two acidic functional groups of the glutamate in contrast to that of the active drug, which is more lipophilic. This was the rationale for the synthesis of a series of L-glutamyl amides of nitrogen mustards derived from 4-aminobenzoic acid, notably the CMDA prodrug (**XIII**) (Mann *et al*, 1990; Springer *et al*, 1990), and after cleavage of the amide bond by CPG2 the resulting drugs become more reactive. Further studies of this ADEPT system demonstrated its ability to achieve both the ablation of chemo-resistant CC3 choriocarcinoma human tumour xenografts in nude mice (Springer *et al*, 1991) and growth delays of OvCa-433 ovarian human tumour xenografts (Sharma *et al*, 1994).

Pilot scale ADEPT clinical trials demonstrated the feasibility of the approach (Bagshawe *et al*, 1991, 1994; Bagshawe, 1995; Napier *et al*, 2000). However, the system was complex, the antibody–enzyme conjugate difficult to make reproducibly, and the antibodies and enzymes were immunogenic, preventing more than two or three administrations even with immunosuppressive therapy. These problems have been addressed by making a genetic fusion protein of a single chain Fv (sFv) (Begent *et al*, 1996) antibody with CPG2 (Bhatia *et al*, 2000). The design features of this molecule include phage library-derived sFv antibody of appropriate affinity linked to CPG2 enzyme, low immunogenicity, dimeric structure, stability *in vitro* and *in vivo*, therapeutic levels of enzyme localising to tumour, clearance from normal tissues in both mice and man due to glycosylation (by fermentation in *Pichia pastoris*), low toxicity, and prodrug activation with therapeutic activity.

All of these features have now been achieved. The fusion protein, MFE23-CPG2 gives tumour to normal ratios of 100–1000 to 1 in an animal tumour model without a separate clearance system. Human colon carcinoma xenografts show tumour regression without significant toxicity and this fusion protein is now being used with the bis-iodo-phenol mustard prodrug (ZD2767P, **XIV**) in a Cancer Research UK Phase I/II trial. Further bioinformatics-led engineering of the enzyme and antibody are in progress to reduce immunogenicity (Boehm *et al*, 2000; Spencer *et al*, 2002).


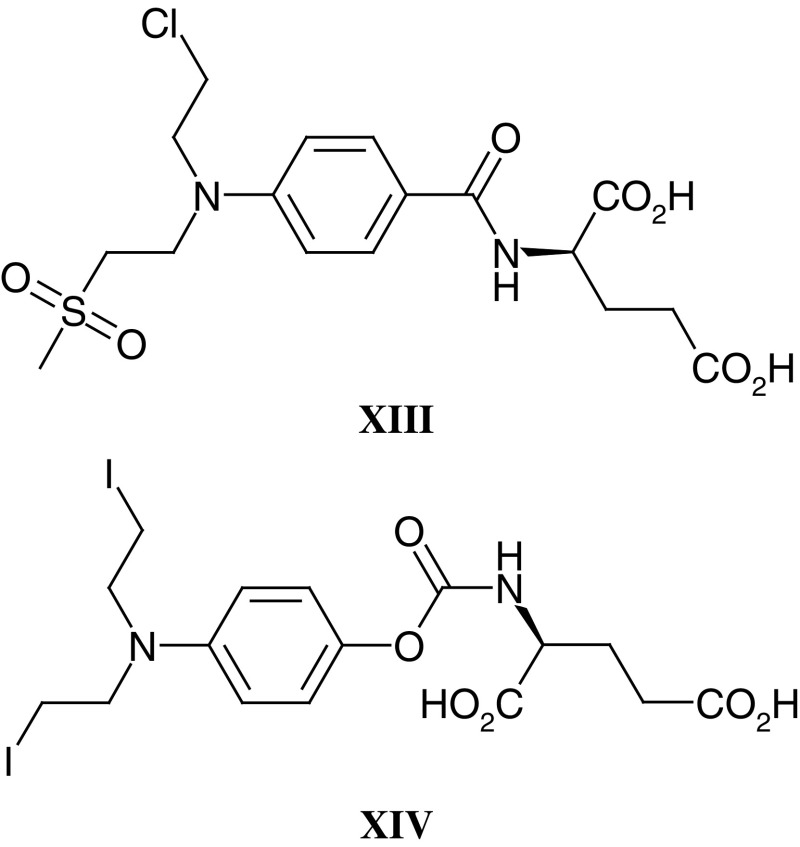


Recent advances in biotechnology combined with the original work performed by Tom Connors on nitrogen mustard drugs now show great promise for development of a practical ADEPT system for the first time.

**CB1954 : from the Walker tumour to NQO2 and gene-directed enzyme prodrug therapy (GEDPT)**—Richard Knox and David Kerr

A common theme throughout Tom Connors' research career was the search for antitumour specificity, and CB1954 (**XV**) is a rare example of a compound that shows outstanding tumour selectivity. Whilst chemically only a monofunctional alkylating agent (by virtue of its single aziridine function), CB1954 exhibited a dramatic curative and highly selective action against the Walker 256 rat tumour with the highest therapeutic index of any compound studied (TI=70) (Cobb *et al*, 1969; Connors and Melzack, 1971). It was subsequently shown that Walker cells could bioactivate CB1954 by aerobic reduction of the 4-nitro group to the corresponding hydroxylamine, which rapidly acylated to a bifunctional alkylating agent (Roberts *et al*, 1986; Knox *et al*, 1988b, 1991). The enzyme responsible for reducing CB1954 in rat tissues is DT-diaphorase (NQO1, NAD(P)H dehydrogenase (quinone) (Knox *et al*, 1988a); however, the human form of DT-diaphorase metabolises CB1954 much less efficiently than rat DT-diaphorase (Boland *et al*, 1991), and even cells that are high in human DT-diaphorase are insensitive to the drug (Boland *et al*, 1991; Mehta *et al*, 1999).


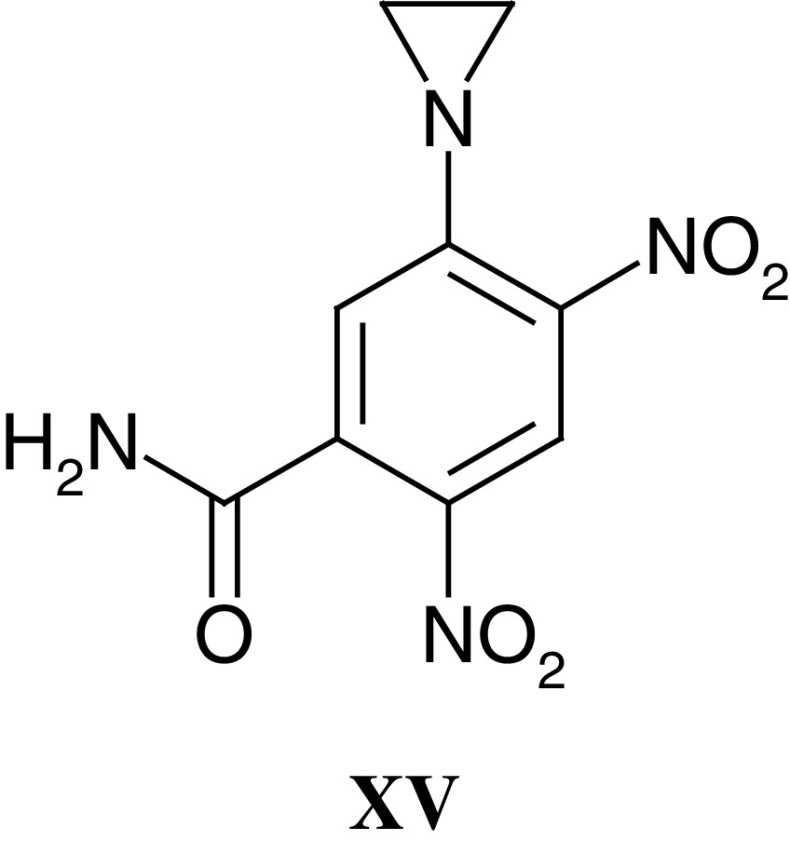


Given the limited ability of human NQO1 to activate CB1954, the drug became an ideal candidate for gene therapy in which the gene delivered to the tumour is an NQO1 that can activate CB1954. *Escherochia coli* nitroimidazole reductase (nitroreductase, NTR) can bioactivate CB1954 much more efficiently than even rat DT-diaphorase (Knox *et al*, 1992) and human tumour cells transduced with retroviral vectors to express this enzyme are very sensitive to the drug (Bridgewater *et al*, 1995; Bailey *et al*, 1996; Green *et al*, 1997; McNeish *et al*, 1998). It was shown that NTR-expressing cell lines are 500–2000-fold more sensitive to CB1954 than parental cell lines (Green *et al*, 1997; McNeish *et al*, 1998). Importantly, a strong bystander effect operates and, with only 5% of cells transfected, high levels of total cell kill (>90%) were observed (McNeish *et al*, 1998) with similar efficacy *in vivo* (Weedon *et al*, 2000).

Phase I clinical trials, initially with a viral gene vector and CB1954 being administered as single agents have been performed, with the intention of combining the agents once adequate tumour expression of NTR has been documented following intratumoral virus injection. CB1954 was administered by i.v. injection every 3 weeks, or i.p. followed by 3-weekly i.v. injections, up to a maximum of six cycles. The CB1954 dose was escalated from 3 to 37.5 mg m^–2^, at which point dose-limiting diarrhoea and hepatic toxicity was seen. CB1954 was well tolerated at a dose of 24 mg m^–2^, and sufficient serum/peritoneal levels are achieved for an enzyme–prodrug approach to be feasible (Chung-Faye *et al*, 2001).

More recently, NTR-expressing replication-deficient adenovirus (under the control of the CMV promoter) has entered trial in patients with resectable liver cancer. Virus is administered 2–4 days preoperatively by intratumoral injection and the kinetics of virus distribution, NTR expression, virus shedding and the appearance of neutralising antibodies is being assessed. So far, 12 patients have been entered and virus dose has been escalated from 10^8^ to 10^11^ virus particles without any serious toxicity.

As an alternative approach to the use of CB1954, it has recently been shown that there is potent endogenous CB1954-reducing activity in human tumour cells (Wu *et al*, 1997), although reduction is only detected in the presence of the cosubstrate dihydronicotinamide riboside (NRH). The latent activity is due to an endogenous enzyme designated NAD(P)H quinone oxidoreductase 2 (NQO2) (Wu *et al*, 1997), which can be considered as a human NRH-dependent nitroreductase (Knox *et al*, 2000a). A simple reduced pyridinium derivative designated EP-0152R can, like NRH, act as cosubstrate for NQO2, and CB1954 is an effective antitumour agent when given with EP-0152R *in vivo* against experimental human tumour xenografts (Knox *et al*, 2000b).

Given the provenance of CB1954, the apparently favourable distribution of NQO2 in certain tumour types and the lack of acute toxicity of EP-0152R, NQO2 represents a promising novel target for antitumour prodrug therapy. A Phase I clinical trial combining CB1954 with EP-0152R is due to start in 2003. CB1954 has been described as ‘a drug in search of a human tumour to treat’ (Workman *et al*, 1986a, 1986b). The GDEPT and NQO2 studies described above may allow this vision to be realised.

**Polymeric drug delivery**–Ruth Duncan and Jim Cassidy

Alongside attempts to find novel tumour-specific targets, a variety of technologies have been developed for the improved delivery or targeting of existing and new drugs to tumours. Increased localisation in tumour tissue (>10-fold) should theoretically overcome drug resistance. Furthermore, ‘steering’ chemotherapy away from normal tissues should reduce toxicity.

The use of polymer–drug conjugates for tumour targeting combines the versatility of polymer and organic (peptide and medicinal) chemistry to allow optimisation of the macromolecular prodrug design. It also takes advantage of passive tumour targeting as the result of the enhanced permeability of angiogenic vessels, the enhanced permeability and retention (EPR) effect (Matsumura and Maeda, 1986). Both conventional chemotherapeutics and drugs designed to interact with novel targets can be polymer-bound. In theory, the construct can also incorporate ligands to facilitate receptor-mediated tumour targeting (Duncan and Kopecek, 1984; Duncan *et al*, 1986; Duncan, 1992; Duncan and Spreafico, 1994; Brocchini and Duncan, 1999). The original model proposed by Ringsdorf (1975) envisaged a hydrophilic polymer backbone chosen to aid drug solubilisation, a biodegradable polymer–drug linker designed to ensure stability in the circulation, and a targeting ligand to promote tumour-specific delivery. Required conjugate characteristics are now well established. The polymer backbone must be nontoxic and nonimmunogenic, must have sufficient drug-carrying capacity and preferably be biodegradable to ensure eventual elimination. If the backbone is not degradable the molecular weight should be limited to <40 000 Da to ensure eventual renal elimination.

Polymer conjugation is also an attractive opportunity to solubilise poorly water-soluble drugs (Caiolfa *et al*, 2000). Drug conjugation limits cellular uptake to the endocytic route, and once internalised the conjugate is trafficked via the endosomal compartments (acid pH 6.5–5.5) to lysosomes and thereby exposed to pH ∼5.0 and an array of lysosomal hydrolases, that is lysosomotropic delivery. The polymer–drug linkage must be completely stable in transit, not degrading too rapidly in the bloodstream or during renal excretion—the primary route of elimination. Extensive studies involving HPMA copolymers bearing libraries of model peptidyl linkers (reviewed in Duncan, 1992) enabled design of the tetrapeptide linker Gly-Phe-Leu-Gly cleaved by the lysosomal cysteine proteases originally used for doxorubicin conjugation (**XVI**–PK1 (FCE28068)). Recently, a combination of a peptide linker and a terminal ester bond was used to synthesise an HPMA copolymer conjugate of paclitaxel and camptothecin (Meerum Terwogt *et al*, 2001) and a variety of platinum-binding ligands including a pendant diamine, malonate and simple carboxylate were used to generate a family of HPMA copolymer platinates (Gianasi *et al*, 1999). Whereas the malonate and carboxylate released biologically active platinum species hydrolytically, the HPMA copolymer-Gly-Phe-Leu-Gly-ethylenediamine-platinate required enzymatic cleavage.


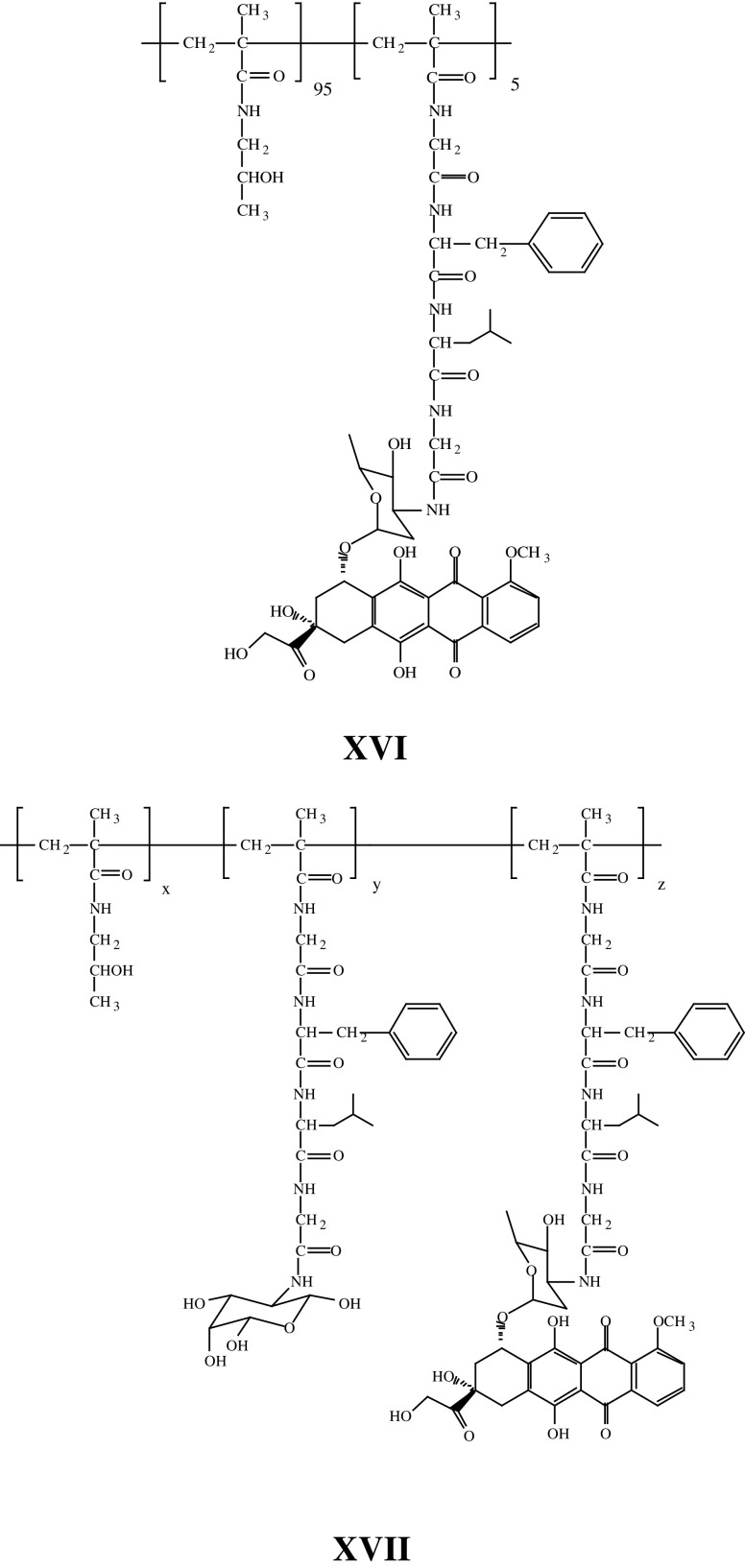


Tumour-specific (or tumour-enhanced) receptors appear to offer the most elegant opportunity for targeted delivery. Although a vast array of putative tumour targeting residues have been investigated, so far the only ‘targeted’ conjugate to enter clinical testing is an HPMA copolymer–doxorubicin conjugate containing, additionally, galactosamine (**XVII**, PK2 (FCE28069)). This conjugate was designed (Duncan *et al*, 1983, 1986) to target the asialoglycoprotein receptor present on normal hepatocytes and hepatocellular carcinoma with the hope of improving treatment of primary and secondary liver cancer. Liver targeting of this conjugate has been verified by clinical gamma camera imaging (Seymour *et al*, 2002).

The Phase I trial of PK1 was performed in 1994–1995 (Vasey *et al*, 1999). It was possible to escalate the doses of doxorubicin to levels four-fold higher than those of free doxorubicin. Importantly, no polymer backbone-related toxicity was encountered and no cardiac effects were noted despite the high levels of doxorubicin-equivalents being given. Pharmacokinetic studies confirmed that PK1 was acting as a depot within the circulation with terminal half-lives of 93 h for total doxorubicin and 108 h for doxorubicin itself. Studies with ^131^I-labelled PK1 showed evidence of tumour drug uptake in some patients. Notably, anticancer activity was demonstrated in this trial, including patients with anthracycline-resistant breast cancer. Overall, the trial addressed many of the outstanding questions in the field and opens the area to new polymeric agents.

PK1 has subsequently undergone Phase II evaluation in colorectal cancer (as an example of an anthracycline-resistant cancer), in non-small-cell lung cancer, and in breast cancer patients (both anthracycline naive and anthracycline resistant). These studies are currently being concluded and it is clear that PK1 has no activity in colorectal cancer but some activity in NSCLC. Further studies are required to elucidate the magnitude of the activity of PK1 in NSCLC in comparison to that of free doxorubicin.

In a Phase I trial, PK2 was shown to produce significant targeting to both normal liver and hepatic tumours with partial clinical responses (Seymour *et al*, 2002). A Phase II evaluation of PK2 in hepatocellular carcinoma is planned.

Phase I/II/III studies are also ongoing with the polyglutamate (PGA)-paclitaxel conjugate CT-2103 (Bolton *et al*, 2002; Sabbatini *et al*, 2002). The drug is linked through the 2′ position via an ester bond to a biodegradable PGA polymer of ∼80 000 Da molecular weight. A significant number of patients have displayed partial responses or stable disease (ovarian, colorectal, NSCLC) both with CT-2103 alone and when combined with cisplatin or carboplatin and patients with paclitaxel-resistant ovarian cancer have shown responses. Lastly, HPMA copolymer-platinate (AP5280), PEG-camptothecin and polysaccharide–camptothecin polymeric antitumour agents have also commenced Phase I trials, and the results are awaited with interest.

Tom Connors strongly supported the development of a completely new class of antitumour compounds in the form of polymer–drug conjugates. Thanks to his support, the field is now well-established with 11 compounds, plus two imaging agents, in clinical trials.

## SELECTED ACTIVITIES AND ACHIEVEMENTS OF THE CANCER RESEARCH UK PHASE I/II COMMITTEE

**Chemistry-driven drug discovery–*N*-methylformamide to Phortress**–Malcolm Stevens and Edward Newlands

Among the outstanding successes of the Phase I/II Clinical Trials Committee as a mechanism for the evaluation of new anticancer agents and diagnostics, is the development of the methylating agent temozolomide (**I**–Temodal®) (Stevens and Newlands, 1993; Newlands *et al*, 1997). This drug arose as a result of multidisciplinary research in which interaction between medicinal chemists and pharmacologists was critical. Temozolomide was selected from a group of imidazotetrazine derivatives synthesised by Stevens and colleagues. The lead compound mitozolomide had shown unpredictable and severe myelosuppression in the clinic (Newlands *et al*, 1985) whereas in murine toxicology studies temozolomide had no dose limiting myelosuppression and was also shown to be schedule dependent in its antitumour activity. The early clinical trials of temozolomide, conducted by the Phase I/II Committee, were performed with extensive laboratory input, pharmacokinetic studies in particular, to establish the optimum route, schedule and dose of administration (Newlands *et al*, 1992).

The Phase I/II Committee looked at neoadjuvant temozolomide given postsurgery and preradiotherapy in patients with high grade gliomas. *In vitro* experiments confirmed that temozolomide was additive when combined with radiation in glioma cell lines in tissue culture. A continuous schedule of temozolomide was developed to be given throughout the patient's radiotherapy; this was shown to be safe and active in patients with glioma.

The mechanism of action of temozolomide was studied using ^11^C-labelled temozolomide PET scanning and pharmacology (Newlands *et al*, 1997) confirming that the ^11^C label in the methyl position was retained by glioma tissue in patients. In contrast, a tracer dose of ^11^C-temozolomide, labelled in the carbonyl position largely appeared in the patient's breath as exhaled CO_2_.

Since Schering Plough took over the development of temozolomide they have performed registration trials in patients with high-grade glioma leading to the licence of temozolomide in the USA and Europe in 2000. There is considerable interest in looking at more extended treatment delivery schedules, drug combinations and combining temozolomide with radiation in patients with brain metastases from tumours outside the central nervous system. Of particular interest is the recent EORTC randomised trial comparing surgery and radiotherapy in one arm, with surgery, radiotherapy and continuous temozolomide in the second arm for patients with glioblastomas. In total, 573 patients were accrued in approximately 24 months to March 2002 and the results are eagerly awaited. Experimentally, the antitumour activity of temozolomide is increased in the presence of some DNA repair inhibitors, in particular the alkyltransferase inhibitor PaTrin 2, and clinical trials of temozolomide plus Patrin 2 are also ongoing.

In addition to temozolomide, the group led by Malcolm Stevens, at Aston University and at the University of Nottingham, have been responsible for the development of four clinical trial candidates. These are *N*-methylformamide (**XVIII**) (McVie *et al*, 1984), mitozolomide (**XIX**) (Newlands *et al*, 1985), MZPES (**XX**) (Stuart *et al*, 1989) and Phortress (**XXI**) (Bradshaw *et al*, 2001, 2002). The latter compound exemplifies perfectly how a novel chemical with interesting pharmacological properties (ie exquisite selectivity for a small number of tumour cell lines *in vitro*) can be used to unravel fascinating biology (ie binding to the arylhydrocarbon receptor, cytochrome *P*450 1A1 induction, and activation to a highly potent DNA-reactive species), which ultimately results in a clinical trial candidate. The clinical trial with Phortress, planned for 2003, will contain the full spectrum of translational research aimed at confirming the mechanism of action in patients that has been comprehensively defined by Tracey Bradshaw and colleagues in their preclinical studies (Chua *et al*, 2000; Bradshaw *et al*, 2001; Loaiza-Perez *et al*, 2002).


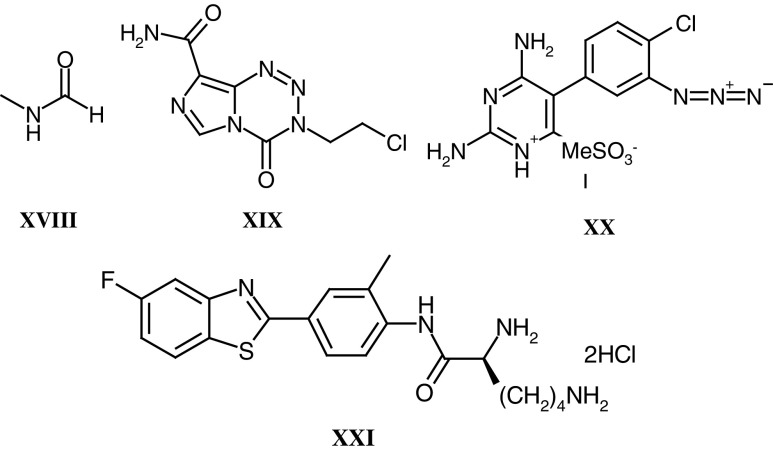


**Platinum complexes: cisplatin to BBR3464**–Hilary Calvert and Eve Wiltshaw

The platinum complexes cisplatin (**XXII**) and carboplatin (**XXIII**) are among the most successful anticancer agents developed. Their curative activity is seen in testicular teratoma and certain paediatric solid tumours, as well as significant effects against a broad spectrum of more common malignancies, notably ovarian cancer, head and neck cancer, and small cell and nonsmall cell lung cancer. Tom Connors was among the first to study the activity of platinum complexes in preclinical tumour models (Connors *et al*, 1972). Sir Alexander Haddow, recognising the potential importance of this new class of drugs, encouraged the initiation of clinical trials with cisplatin at the Royal Marsden Hospital in 1972 by Eve Wiltshaw. These early clinical trials identified activity in patients with relapsed ovarian cancer although the toxicities were also severe–profuse nausea and vomiting, and renal failure. The advent of 5-hydroxytrypamine receptor type 3 antagonists and the use of hydration/diuresis has largely controlled these side effects, respectively. However, an alternative approach was initiated by Tom Connors and Ken Harrap which resulted in the identification of carboplatin as an equiactive yet non-nephrotoxic platinum complex. The first Phase I trial of carboplatin was completed in 1981 and this identified dose-limiting haematological toxicity, as well as early signs of activity, but failed to detect any significant renal side effects (Calvert *et al*, 1982). The most important difference between cisplatin and carboplatin is the 30-fold reduced reactivity, which allows renal excretion without renal damage, as well as reduced emesis and neurotoxicity. However, whereas carboplatin produces no significant nephrotoxicity, renal function does have a marked effect on the haematological toxicity of the drug. Subsequent pharmacokinetic studies led to the development of renal function-based carboplatin dosing using the ‘Calvert’ formula (Calvert *et al*, 1989). Using this formula, the absolute dose (in mg) required for each patient is calculated from the desired target area under the free carboplatin plasma concentration *vs* time curve (AUC–mg ml^–1^ min) and the patient's absolute glomerular filtration rate (GFR–ml min^–1^, preferably measured by an isotopic method and not from serum creatinine alone):

Carboplatin dose(mg) = Target AUC × (GFR(ml min^−1^) + 25)

Despite the success of cisplatin, and subsequently carboplatin in which Cancer Research UK scientists played no small part, the complexes suffered from the problems of inherent and acquired resistance that has beset all forms of cytotoxic chemotherapy. In a programme led by Ken Harrap at the Institute of Cancer Research, London, extensive studies were undertaken to identify Pt-complexes with a broader spectrum of activity than cisplatin and carboplatin, and/or activity in disease resistant to these agents. Two compounds derived from this programme, JM216 (**XXIV**) and AMD473 (**XXV**) were taken into Phase I clinical trials by the Phase I/II Committee. These trials led to further research with the objective of demonstrating activity in patients with Pt-resistant tumours, or tumour types where Pt-complexes are not generally considered active. Of over 15 complexes studied in clinical trials, only oxaliplatin has a proven clinical efficacy profile significantly different from that of the first-generation complexes (cisplatin and carboplatin), in this case activity in colorectal cancer.


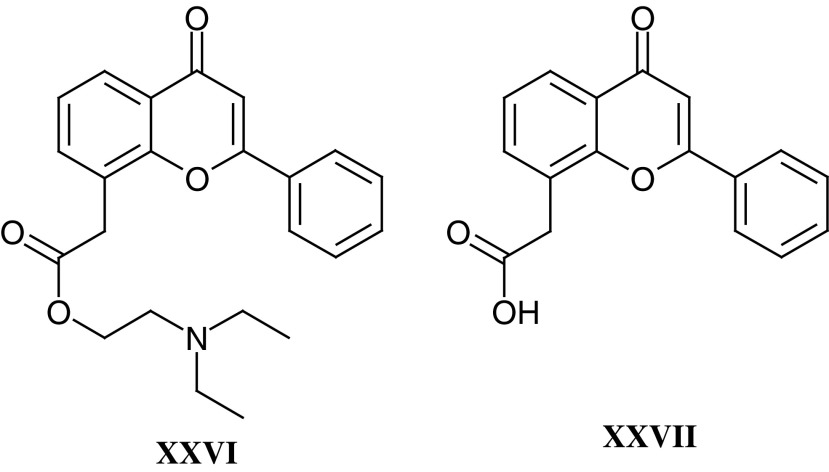


Most recently, the Phase I/II Committee has completed studies with the triplatinum complex BBR3464 which has preclinical activity in a panel of 18 xenograft models that was clearly superior to that of cisplatin. In particular, BBR3464 was active against tumours with mutant p53. Despite these clinical data there was only one partial response in 19 ovarian cancer patients who were platinum refractory, which again brings into question the value of preclinical tumour models when used in a screening as opposed to a mechanistic mode.

Overall, from the early days of cisplatin at the Royal Marsden Hospital through the development of carboplatin to more recent clinical trials with second/third-generation complexes, Cancer Research UK scientists and the Phase I/II Committee have played a major role in the development of this important class of drugs.

**Antivascular agents**–Mike Bibby and Gordon Rustin

During tumour angiogenesis endothelial cell division increases dramatically giving rise to an exploitable feature for therapy (Denekamp, 1982). A key advantage of targeting the tumour vasculature is that endothelial cells are genetically stable and hence are less likely to develop drug resistance. Furthermore, the normal complement of apoptosis-related genes should still be functional, which also, in theory, makes the endothelial cell an attractive target. There are two principal therapeutic approaches by which the tumour vasculature can be targeted. Firstly, antiangiogenic strategies, which involve interrupting the actual process of angiogenesis, and secondly antivascular approaches, which aim to damage the existing vessels within tumours. The Phase I/II Committee has now evaluated four antivascular drugs.

Flavone acetic acid ester (**XXVI**) (NSC 293015, LM985) emerged as a lead compound from a series of flavonoids from Lyonnaise Industrielle Pharmaceutique (Lipha) that were screened by the National Cancer Institute (NCI), and as a result of solid tumour activity in preclinical models LM985 was selected by the Phase I/II Committee. However, drug-associated hypotension was encountered in Phase I trials with LM985 (Kerr *et al*, 1985). The free acid flavone acetic acid (**XXVII**) (NSC 346512, LM975, FAA) was selected and studied as a back-up (Kerr *et al*, 1987), as it was responsible for the antitumour effects seen in mice (Double *et al*, 1986; Bibby *et al*, 1987a).

*In vitro* studies of FAA (Bibby, 1991; Bibby and Double, 1993) demonstrated that very high concentrations or long exposure times, in excess of those achieved *in vivo*, were necessary for direct cytotoxicity (Bibby *et al*, 1987) and indicated that an established blood supply to the tumour was necessary for response (Double *et al*, 1986). It became clear that the vascular component was of major importance for the response of tumours in mice (Bibby *et al*, 1989; Hill *et al*, 1989; Zwi *et al*, 1989). In addition, FAA produced significant immune effects (reviewed in Bibby and Double, 1993), and Ching and Baguley (1988) demonstrated that FAA could enhance the lytic effects of peritoneal macrophages *in vitro* against a range of tumour cells. Furthermore, TNF*α* was implicated as a major factor in FAA-induced vascular shutdown, and the haemorrhagic necrosis seen in subcutaneously transplanted tumours treated with FAA had already been likened to effects seen after TNF*α* treatment.

Although these preclinical studies with FAA were exciting, the drug was consistently inactive in Phase II clinical trials (eg Kerr *et al*, 1989), and hence FAA analogues were synthesised in an attempt to identify an agent that reproduced in humans the effects seen in mice. A series of analogues with the structurally related xanthenone chromophore were developed (Atwell *et al*, 1989; Rewcastle *et al*, 1989, 1991a and 1991c), and DMXAA (**VII**) was identified as an agent that produced a similar degree of haemorrhagic necrosis to FAA against the colon tumours but at 10–15-fold lower doses (Rewcastle *et al*, 1991c; Laws *et al*, 1995). As with FAA, DMXAA produced numerous immune effects *in vivo* although these occurred at much lower concentrations. DMXAA was more potent at inducing expression of TNF*α* mRNA than FAA in murine and human cells (Ching *et al*, 1994). A Phase I trial by the Phase I/II Committee has shown, using Dynamic Contrast Enhanced MRI (DCE-MRI), that DMXAA can cause changes consistent with a maintained reduction in blood flow in tumours, but not muscle, in patients (Galbraith *et al*, 2002). Weekly or three-weekly dosing was well tolerated at doses up to 3700 mg m^–2^ and, in addition to the DCE-MRI changes, elevated tumour but not serum TNF levels and plasma serotonin metabolite levels were observed, consistent with preclinical data (M Jameson and GJ Rustin, personal communications). Combination studies with cytotoxic drugs are therefore planned with DMXAA and the combination with paclitaxel is particularly impressive in preclinical studies (Siim *et al*, 2003).


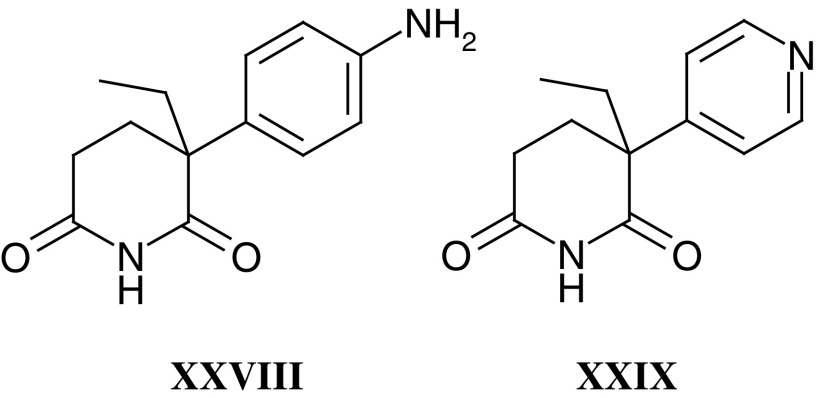


The fourth antivascular agent to be studied by the Phase I/II Committee is combretastatin A4 phosphate (**VIII**) (CA4P). The combretastatins are a family of compounds derived from the African tree *Combretum caffrum* (Pettit *et al*, 1987, 1989). CA4P is a relatively simple stilbene that has been shown to interact with tubulin at or near the colchicine-binding site, resulting in the inhibition of tubulin assembly and therefore disruption of microtubular function (McGown and Fox, 1989). Treatment with combretastatin A4, and its more soluble prodrug CA4P (Pettit *et al*, 1995), results in vascular shutdown within the tumour causing massive haemorrhagic necrosis (Dark *et al*, 1997; Grosios *et al*, 1999). In animals, CA4P treatment results in significant antitumour effects at less than one-tenth of the MTD (Dark *et al*, 1997). Early clinical studies by the Phase I/II Committee have shown that CA4P is well tolerated at doses below 52 mg m^–2^ but that dose-limiting neurological, cardiac and vascular effects, including tumour pain, are seen at higher dose levels (Stevenson *et al*, 2000). Again a significant reduction of tumour blood flow has been demonstrated by DCE-MRI and PET, and evidence of biological activity has been seen (Galbraith *et al*, 2000). CA4P combination studies with both cytotoxic agents and radio-labelled tumour selective antibodies are planned.

The studies with antivascular agents demonstrated the ability of the Phase I/II Committee to undertake mechanistic early clinical trials in which pharmacodynamic end points are a key component. Such studies are essential when the target is tumour blood vessels, as preclinical studies showed minimal tumour size reduction when vascular targeting drugs were given alone. To make decisions regarding further development, especially in combination studies, it is essential to know whether these drugs are affecting tumour blood flow in patients.

**Antiendocrine agents**–Michael Jarman and Charles Coombes

The preclinical and clinical development of antiendocrine agents by the Phase I/II Committee has variously involved collaborations between the Institute of Cancer Research, Royal Marsden Hospital, Imperial College School of Medicine (ICSM) and Charing Cross Hospital. The approach taken, that is metabolism-directed drug discovery, began in collaboration with Tom Connors at the Institute of Cancer Research in the early 1970s, and was later applied to tamoxifen, targeting the oestrogen receptor, and aminoglutethimide (**XXVIII**), which inhibits the aromatase enzyme catalysing the final step in the synthesis of oestrogens. Aminoglutethimide inhibits this cytochrome *P*450 enzyme via its basic amino group, but metabolism by N-acetylation or N-hydroxylation inactivates it. This inactivation was circumvented in the pyridyl analogue Rogletimide (**XXIX**) (formerly pyridoglutethimide). Unlike aminoglutethimide, Rogletimide selectively inhibited aromatase. In the Phase I trial on 10 patients it reduced circulating oestradiol by 50% at doses above 200 mg b.d. (Dowsett *et al*, 1991). However, rogletimide also induced its own metabolism, and plasma half-life fell with repeated dose (Haynes *et al*, 1991), so that potency was compromised. Aromatase inhibitors of much greater potency have now superseded Rogletimide. One of these, 4-hydroxyandrosteniodone (**II**), was synthesised and tested under the auspices of the Phase I/II Committee back in 1982 and was subsequently licensed to Novartis. It was available in many countries until this year, when potent third-generation compounds became available.

Idoxifene (**XXX**) was developed in response to a perceived need to block the metabolism of tamoxifen to its 4-hydroxy derivative. Though binding 100-fold more strongly to the target oestrogen receptor, 4-hydroxytamoxifen is rapidly cleared via its glucuronide. The 4-iodo substituent in idoxifene prevents 4-hydroxylation and confers an improved binding affinity compared with tamoxifen. In a Phase I trial on 20 patients (Coombes *et al*, 1995), it had a longer half-life than tamoxifen, and evidence of partial response following relapse on tamoxifen. However a Phase II trial, comparing idoxifene and tamoxifen, both at 40 mg o.d., in 48 postmenopausal patients, who had previously received tamoxifen, showed oestrogenic side effects and no evidence that idoxifene was better in this setting.

Abiraterone (**XXXI**) is a potent inhibitor of the enzyme, cytochrome *P*450-17*α*, that mediates the biosynthesis of the immediate precursors of the active androgenic steroids testosterone and 5 *α*-dihydrotestosterone (Potter *et al*, 1995). It could therefore inhibit both testicular and adrenal androgen biosynthesis, producing maximal androgen ablation in the treatment of hormone-dependent prostate cancer. A Phase I trial (principal investigators Dr I R Judson and Dr D Dearnaley) evaluating the prodrug abiraterone acetate in three settings looks encouraging; (a) single dose in castrate patients, (b) single dose in noncastrate patients, (c) multiple dose in noncastrate patients.


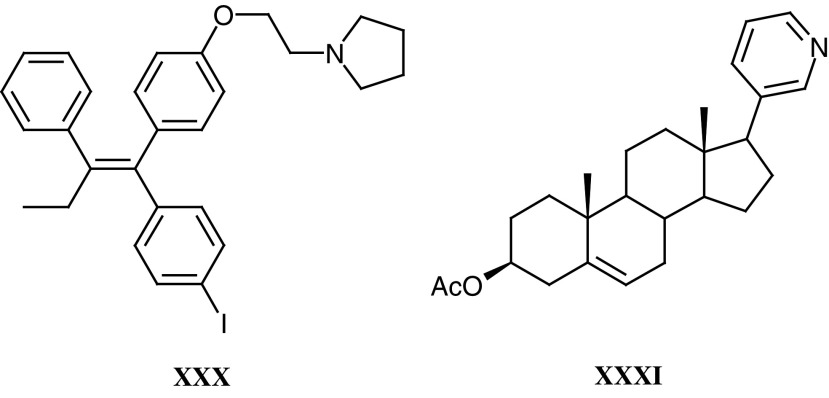


**Immunotherapies**–Peter Amlot and Robert Hawkins

The Phase I/II Committee also has taken on active interest in the development of immunotherapies, including direct antibody-based treatments and antibody, DNA and cell-based vaccines.

The advances made possible by the development of monoclonal antibody biotechnology has allowed the development of reagents with exquisite specificity for tumour-associated antigens. The antibodies can be used either in native form, for example, anti-CD5, CD20, CD21 and CD52 for the treatment of haematological malignancies, or conjugated to ribosome-inactivating toxins such as ricin or saporin, to form immunotoxins. The Phase I/II Committee has recently sponsored trials with an anti-CD19 antibody and an anti-CD38 antibody conjugated to saporin for the treatment of childhood acute lymphoblastic leukaemia and multiple myeloma, respectively.

The immunogenicity of murine monoclonal antibodies necessitated the development of humanised or chimeric antibodies. However, the over-riding problems of toxicity and immunogenicity remain significant issues. In particular, vascular leak syndrome (VLS) is a complex and difficult-to-manage side effect of immunotoxins. The mechanism underlying this effect is thought to be the interaction between the toxin moiety and the vascular endothelium, and a satisfactory way of overcoming this problem has yet to be defined. Nonetheless the CD22-*pseudomonas* exotoxin fusion protein CD22-PE is one of the most effective treatments for hairy cell leukaemia (Kreitman *et al*, 2001). However, the more widespread use of immunotoxins will require the resolution of toxicity and immunogenicity issues, and in the meantime alternative antibody therapies such as ADEPT (see above) may offer more promise.

While vaccination has played a major role in the control or even eradication of infectious diseases, it has yet to make a major impact on cancer. Successful vaccination requires a number of components; namely, a viable tumour antigen, a feasible vaccine methodology and an understanding of the type of immunity required to control the tumour. A large number of tumour-associated target antigens have been identified and these include:

- Oncofoetal antigens: for example carcinoembryonic antigen.
- Differentiation antigens: for example gp100 in melanoma.
- Mutated gene products: for example p53 and ras.
- Viral gene products: for example those produced by human papilloma virus (cervical cancer), Epstein–Barr virus (Burkitt's lymphoma) and hepatitis B virus (hepatocellular carcinoma).
- Idiotypic epitopes: for example Ig idiotypes (B-cell lymphoma and multiple myeloma) and TCR idiotypes (T-cell lymphoma).

Vaccination methods include the use of proteins, plasmids, viruses, dendritic cells and combinations of these approaches. The Phase I/II Committee has had a particular interest in the evaluation of vaccines to exploit idiotypes, and studies with both antibody and plasmid vaccination approaches were undertaken (Hawkins *et al*, 1997). For example, in an ongoing trial of a plasmid vaccine for the treatment of patients with B-cell lymphoma, the gene for fragment C of tetanus toxin is fused with the lymphoma idiotype gene from individual tumours and the fusion gene given as repeated immunisations while the patient is in complete remission. Doses of 0.5–2.5 mg of plasmid DNA have been given and both antitetanus and antiidiotype responses are being measured.

Looking to the future, viral DNA delivery, as opposed to naked plasmid gene administration, may be more efficient (Armstrong *et al*, 2002), either used alone or in combination with dendritic cells. Recent studies with protein-loaded dendritic cells have demonstrated clinical activity (Timmerman *et al*, 2002), and dendritic cells have the advantage of efficient antigen uptake and presentation, as well as the expression of the full range of accessory molecules required for an efficient immunodynamic effect.

## OVERVIEW AND CONCLUSIONS

This article has reviewed only selected aspects of the activities of the Phase I/II Clinical Trials Committee since its creation in 1980, and a full list of all the agents selected for clinical trials, as of November 2002, is given in Table 2

**Table 2:** Projects undertaken by the Phase I/II Clinical Trials Committee 1980–2002

**Compound and mechanism of action**	**CR-UK studies**	**Current status of agent (not all CR-UK trials)**	**Reasons for discontinuation**
*Cytotoxic drugs*			
1069-C85–tubulin binding agent	Phase I	Discontinued after Phase I	Unacceptable toxicity
AG2034–antipurine antifolate	Phase I	Discontinued after Phase I	Superseded by improved analogue AG2037
AMD473 (ZD0473)–platinum complex	Phase I/II	Phase II studies ongoing	
Amphetinile–tubulin binding agent	Phase I	Discontinued after Phase I	Unacceptable toxicity
Amsalog–topoisomerase inhibitor (m-AMSA derivative)	Phase I (oral)	Discontinued after Phase I	Poor oral bioavailability
AQ4N–reductively-activated topoisomerase inhibitor	Phase I	Phase I study ongoing	
BBR3464–platinum complex	Phase II	Discontinued after Phase II	Lack of activity
Biantrazole (DUP941/CI941)–topoisomerase inhibitor	Phase I/II	Registered drug now withdrawn	
BZQ–reductively-activated alkylating agent	Phase I	Discontinued after Phase I	Unacceptable toxicity
C6G mustard–carbohydrate-targeted alkylating agent	Phase I	Discontinued during Phase I	Lack of potency
CB10-277–methylating agent (DTIC analogue)	Phase I/II	Discontinued after Phase II	Lack of activity
Clomesone–alkylating agent	Phase I	Discontinued after Phase I	Unacceptable toxicity
DACA–topoisomerase inhibitor	Phase I	Discontinued after Phase II	Lack of activity
Didox–ribonucleotide reductase inhibitor	Phase I	Discontinued after Phase I	Unacceptable toxicity
Etoposide phosphate–etoposide prodrug	Phase I	Registered drug (Etopophos)	
JM216 (satraplatin)–platinum complex	Phase I	Phase III studies ongoing	
Methane dimethane sulphonate–alkylating agent	Phase I	Discontinued after Phase I	Unacceptable toxicity
Mitozolomide–chloroethylating agent	Phase I/II	Discontinued after Phase II	Superseded by improved analogue temozolomide
MZPES–nonclassical dihydrofolate reductase inhibitor	Phase I	Discontinued after Phase II	Unacceptable toxicity/lack of activity
Nolatrexed (AG337)–thymidylate synthase inhibitor	Phase I	Phase III study ongoing	
RH1–reductively activated alkylating agent	Phase I	Phase I planning ongoing	
Rhizoxin–tubulin binding agent	Phase II	Discontinued after Phase II	Lack of activity
SJG-136–DNA sequence selective minor groove binder	Phase I	Phase I planning ongoing	
Temozolomide–methylating agent	Phase I/II	Registered drug (Temodal)	
Trimelamol–preactivated methylmelamine	Phase I/II	Discontinued after Phase II	Problems with manufacture
			
*Antiendocrine drugs*			
4-Hydroxyandrostenedione–oestrogen synthesis inhibitor	Phase I	Registered drug now withdrawn	
Abiraterone (CB7630)–androgen synthesis inhibitor	Phase I	Phase I completed	
Coumate–oestrone sulphatase inhibitor	Phase I	Phase I planning ongoing	
Idoxifene–anti-oestrogen (tamoxifen analogue)	Phase I/II	Discontinued after Phase II	Oestrogenic side effects
Rogletimide - oestrogen synthesis inhibitor	Phase I	Discontinued after Phase I	Poor pharmacokinetics
			
*Agents with novel or unknown mechanism of action*			
17-Allylaminogeldanamycin–HSP90 ATPase inhibitor	Phase I	Phase I study ongoing	
Batimastat (BB94)–matrix metalloproteinase inhibitor	Phase I	Discontinued	Superseded by improved analogue marimastat
Boronphenylalanine–BNCT^1^ reagent	Phase I	Phase I planning ongoing	
Bryostatin 1–protein kinase C modulator	Phase I/II	Phase II studies ongoing	
CB1954 with NQO2 substrate–bioreductive alkylator	Phase I	Phase I planning ongoing	
Combretastatin-A4 phosphate–antivascular agent	Phase I	Phase II studies ongoing	
CT2584–signal transduction inhibitor	Phase I	Discontinued after Phase II	Problems with formulation
CYC202–cyclin-dependent kinase inhibitor	Phase I	Phase II studies planned	
Decitabine–DNA methyltransferase inhibitor	Phase I	Phase I study ongoing	
DMXAA–antivascular agent/cytokine modulator	Phase I	Phase II planning ongoing	
Elactocin–unknown mechanism	Phase I	Discontinued after Phase I	Unacceptable toxicity
FAA–antivascular agent/cytokine modulator	Phase I	Discontinued after Phase II	Superseded by improved analogue DMXAA
GR63178A–unknown mechanism	Phase II	Discontinued after Phase II	Lack of activity
LM985–antivascular agent/cytokine modulator	Phase I	Discontinued after Phase I	Superseded by improved analogue FAA
LY195448–unknown mechanism	Phase I	Discontinued during Phase I	Preclinical activity not confirmed
OSI774–EGF receptor kinase inhibitor	Phase II	Phase II planning ongoing	
Patrin-2–O^6^-alkylguanine alkyltransferase inactivator	Phase I	Phase II planning ongoing	
Penclomidine–unknown mechanism	Phase I	Discontinued after Phase I	Unacceptable toxicity
Phortress–cytochrome *P*450-activated cytotoxin	Phase I	Phase I planning ongoing	
Phyllanthoside–DNA and protein synthesis inhibitor	Phase I	Discontinued after Phase I	Unacceptable toxicity
RSU-1069–reductively-activated radiopotentiator	Phase I	Discontinued after Phase I	Unacceptable toxicity
SDZ 62-434–unknown mechanism	Phase I	Discontinued after Phase I	Lack of compound supply
PSC-833–P-glycoprotein antagonist	Phase I	Discontinued after Phase III	Lack of sufficient activity
SPAG–mitogenic neuropeptide antagonist	Phase I	Discontinued during Phase I	Lack of potency/rapid clearance
SR4554–magnetic resonance hypoxia imaging agent	Phase I	Phase I study ongoing	
SU6668–growth factor receptor kinase inhibitor	Phase I (oral)	Discontinued during Phase I	Problems with capsule manufacture
Suramin–growth factor antagonist	Phase I/II	Phase I/II study ongoing	
TBI-699–DNA repair inhibitor	Phase I	Phase I planning ongoing	
			
*Polymer targeted agents*			
Biotransdox–polymeric doxorubicin formulation	Phase I	Phase II studies ongoing	
CT2103 (Xyotax)–polymeric paclitaxel	Phase I/II	Phase III studies ongoing	
MAG-CPT–polymeric camptothecin	Phase I	Discontinued after Phase I	Unacceptable toxicity
PK1–polymeric doxorubicin	Phase I/II	Phase II studies completed	
PK2–galactose receptor targeted polymeric doxorubicin	Phase I/II	Phase II study planned	
			
*Antibody targeted agents and immunotherapies*			
105AD7–anti-idiotype CD55 vaccine	Phase I/II	Phase II study ongoing	
5T4–epithelial tumour antigen vaccine	Phase I	Phase II studies ongoing	
^131^I-AFP161–alphafoetoprotein imaging agent	Phase I	Discontinued after Phase I	Adequate imaging not achieved
^131^I-A5B7–anti-CEA radioimmunotherapy	Phase I	Phase I planning ongoing	Combination study with CA4P
^99^Tc-A5B7 /^125^I-A5B7 /^131^I-A5B7–CEA imaging agent	Phase I	Discontinued after Phase I	Superseded by MFE23
ADEPT^2^–A5B7 f(ab)_2_-CPG2 with CMDA prodrug	Phase I	Discontinued after Phase I	CMDA superseded by ZD2767P prodrug
ADEPT–A5B7 f(ab)_2_-CPG2 with ZD2767P prodrug	Phase I	Discontinued after Phase I	Conjugate superseded by MFE23-CPG2
ADEPT–MFE23-CPG2 with ZD2767P prodrug	Phase I	Phase I study ongoing	
Anti-lymphoma idiotype DNA vaccine	Phase I	Discontinued after Phase I	Superseded by idiotype/tetanus (LIFTT) vaccine
Anti-lymphoma idiotype/tetanus DNA vaccine (LIFTT)	Phase I	Phase I study ongoing	
BU12-saporin–anti-CD19 immunotoxin	Phase I	Phase I study ongoing	
^67^Cu-C595–anti-MUC-1 radioimmunotherapy	Phase I	Phase I study ongoing	
Chimeric B72.3–anti-colorectal antigen antibody	Phase I	Discontinued after Phase I	Unacceptable immunogenicity
^131^I-CHT25–anti-IL2 receptor radioimmunotherapy	Phase I	Phase I study ongoing	
EBV–Epstein–Barr virus vaccine	Phase I	Phase I planning ongoing	
ICR62–anti-EGF receptor antibody	Phase I	Discontinued after Phase I	Lack of antibody supply
HPV16–L1 capsid protein vaccine	Phase I	Phase I study ongoing	
IL2 gene therapy–immunostimulation for melanoma	Phase I	Discontinued after Phase I	Problems with manufacture
MID/2/40/C–epithelial antigen imaging agent	Phase I	Discontinued during Phase I	Problems with manufacture
^123^I-MFE23–CEA imaging agent	Phase I	Discontinued after Phase I	Led to MFE23-CPG2 ADEPT
MVA.EBNA.1–Epstein–Barr virus vaccine	Phase I	Phase I planning ongoing	
^131^I-NY.3D11–NCAM imaging agent	Phase I	Discontinued after Phase I	Poor localisation
OKT10-saporin–anti-CD38 immunotoxin	Phase I	Phase I study ongoing	
PK45s–polyclonal sheep anti-CEA antibody	Phase I	Discontinued after Phase I	Superseded by A5B7
SWA11–SCLC imaging agent	Phase I	Discontinued after Phase I	Poor localisation
TNF alpha vaccine	Phase I	Discontinued after Phase I	Lack of immune response

. Specific compounds have been described in detail above, but in total 89 agents have been selected, which includes 25 cytotoxic drugs, five antiendocrine agents, 28 molecules with novel or unknown mechanisms of action, five polymeric antitumour drugs and 26 antibody-targeted agents/immunotherapies. With these agents, no fewer than 85 Phase I and 17 Phase II trials have been performed or are planned by the Phase I/II Committee. As appropriate for an academic clinical trials group operating at the clinical-laboratory interface, activity has deliberately been focused on hypothesis-testing clinical trials with, increasingly, pharmacological or immunological end points.

Of the agents selected by the Phase I/II Committee, 23 are currently undergoing Phase I evaluation (ie trials are either open or planned), 36 did not progress beyond Phase I, and 30 proceeded to further clinical investigation. For those which did not progress beyond Phase I trials, the most common reason was unacceptable toxicity. However, it is important to stress that in no case was toxicity unacceptable at the Phase I trial starting dose and the experience of the Committee, with a wide range of agents, is that rodent-only toxicology does provide a safe Phase I trial starting dose (Newell *et al*, 1999). The second most frequent reason for compounds not progressing beyond Phase I trials is that the agent was superseded by an improved analogue or derivative. Thus, while the agent itself did not progress to become a therapeutic, the Phase I trial performed by the Committee was nevertheless an important contribution to cancer drug development. Other reasons for compounds not progressing beyond Phase I trials included poor pharmacokinetics or potency and problems with the supply of the clinical material.

Of the 30 agents that have progressed to clinical evaluation beyond a Phase I trial, 11 are currently undergoing or are scheduled for Phase II studies. For the 19 compounds where Phase II evaluation has been completed, five were shown to be inactive (BBR3464, CB10-277, DACA, GR63178A and rhizoxin), three were superseded by improved derivatives (batimastat, FAA and mitozolomide), two had formulation/manufacture problems (CT2584 and trimelamol), and one (idoxifene) had poor pharmacodynamics. Eight of the agents that entered Phase II trials, either under the auspices of the Phase I/II Committee or elsewhere, had activity at a level that warranted later stage clinical trials. Of these eight drugs, four subsequently became registered therapies (4-hydroxyandrostenedione, biantrazole, etoposide phosphate and temozolomide), and trials with three agents (CT2103, JM216, nolatrexed) are ongoing.

By any standards, the activities of the Phase I/II Committee constitute an impressive achievement in the arena of Phase I/II clinical trials. Overall, the Committee has made a significant contribution to international anticancer drug discovery and development. That this achievement can be traced directly back to the vision and drive of Tom Connors is without question, and all the cancer patients who have benefited, as well as clinical and laboratory scientists who have undertaken the trials, owe Tom an outstanding debt.

As this report illustrates, the achievements of the Phase I/II Committee are already a lasting memorial to Tom Connors. Cancer Research UK and all those involved in developmental therapeutics in the UK now have a duty to ensure that the network created by Tom, Brian Fox and Laszlo Lajtha in 1980 moves forward to even greater success, and in so doing allows patients to benefit directly from the advances in cancer molecular biology that are unleashing a new generation of treatments.

## Appendix I: Contributors

**PL Amlot** Department of Immunology, Royal Free Hospital, Pond Street, London, NW3 2QG, UK; **RHJ Begent** Department of Oncology, Royal Free & University College, Medical School, Rowland Hill Street, London, NW3 2PF, UK; **MC Bibby** Tom Connors Cancer Research Unit, University of Bradford, Bradford, West Yorkshire, BD7 1DP, UK; **AH Calvert** Northern Institute for Cancer Research, University of Newcastle upon Tyne, The Medical School, Framlington Place, Newcastle upon Tyne, NE2 4HH, UK; **J Cassidy** Cancer Research UK Department of Medical Oncology, Cancer Research UK Beatson Laboratories, Garscube Estate, Switchback Road, Bearsden, Glasgow, G61 1BD, UK; **RC Coombes** Cancer Research UK Laboratories, Department of Medical Oncology, Imperial College School of Medicine, Hammersmith Hospital, Du Cane Road, London, W12 0NN, UK; **JA Double** Tom Connors Cancer Research Unit, University of Bradford, Bradford, West Yorkshire, BD7 1DP, UK; **R Duncan** Centre for Polymer Therapeutics, Cardiff University, Welsh School of Pharmacy, Redwood Building, King Edward VII Avenue, Cardiff, CF10 3XF, UK; **RE Hawkins** Department of Medical Oncology, Christie Cancer Research UK, Research Centre, Christie Hospital NHS Trust, Wilmslow Road, Manchester, M20 4BX, UK; **TA Hince** Cancer Research UK, PO Box 123, 61 Lincoln's Inn Fields, London, WC2A 3PX, UK; **M Jarman** 46 Brodrick Road, London, SW17 7DY, UK; **DI Jodrell** Edinburgh Medical Oncology Unit, Western General Hospital, Crewe Road, Edinburgh, EH4 2XU, UK; **IR Judson** Royal Marsden Hospital, 15 Cotswold Road, Belmont, Sutton, Surrey, SM2 5NG, UK; **DJ Kerr** Department of Clinical Pharmacology, University of Oxford, Radcliffe Infirmary, Woodstock Road, Oxford, OX2 6HE, UK; **RJ Knox** Enact Pharma PLC, Building 115, Porton Down Science Park, Salisbury, Wiltshire, SP4 0JQ, UK; **JG McVie** Cancer Intelligence, 4 Stanley Road, Bristol BS6 6NW, UK; **ES Newlands** Department of Cancer Medicine, Imperial College of Science, Technology and Medicine, Hammersmith & Charing Cross Hospitals, Fulham Palace Road, London, W6 8RF, UK; **P Price** Academic Department of Radiation Oncology, Christie Hospital NHS Trust, Wilmslow Road, Withington, Manchester, M20 4BX, UK; **GJ Rustin** Department of Medical Oncology, Mount Vernon Hospital, Rickmansworth Road, Northwood, HA6 2RN, UK; **DS Secher** Research Services Division, University of Cambridge, 16 Mill Lane, Cambridge, CB2 1SB, UK; **JF Smyth** Edinburgh Medical Oncology Unit, Cancer Research Building, Western General Hospital, Crewe Road South, Edinburgh, EH4 2XR, UK; **CJ Springer** Institute of Cancer Research, 15 Cotswold Road, Belmont, Sutton, Surrey, SM2 5NG, UK; **MFG Stevens** School of Pharmaceutical Sciences, University of Nottingham, Cancer Research Laboratories, University Park, Nottingham, NG7 2RD, UK; **IJ Stratford** School of Pharmacy and Pharmaceutical Sciences, University of Manchester, Oxford Road, Manchester, M13 9PL, UK; **E Wiltshaw** Sandmartins, Sandy Lane, Watersfield, West Sussex, RH20 1NF, UK; **P Workman** Cancer Research UK Centre for Cancer Therapeutics, Institute of Cancer Research, 15 Cotswold Road, Belmont, Sutton, Surrey, SM2 5NG, UK; **OC Yoder** National Cancer Institute, Division of Cancer Treatment and Diagnosis, Building 31, Rm 3A44 MSC 2440, Bethesda, MD 20892 2440, USA

## References

[bib1] Aboagye EO, Kelson AB, Tracy M, Workman P (1998) Preclinical development and current status of the fluorinated 2-nitroimidazole hypoxia probe *N*-(2-hydroxy-3,3,3-trifluoropropyl)-2-(2-nitro-1-imidazoly) acetamide (SR-4554, CRC 94/17): a noninvasive diagnostic probe for the measurement of tumour hypoxia by magnetic resonance spectroscopy and imaging, and by positron emission tomography. Anti-Cancer Drug Des 13: 703–730 9755726

[bib2] Aboagye EO, Maxwell RJ, Kelson A, Tracy M, Lewis AD, Graham MA, Horsman M, Griffiths JR, Workman P (1997) Preclinical evaluation of the fluorinated 2-nitroimidazole *N*-(2-hydroxy-3,3,3-trifluoropropyl)-2-(2-nitro-1-imidazolyl) acetamide (SR-4554) as a probe for the measurement of tumor hypoxia. Cancer Res 57: 3314–3318 9242466

[bib3] Adams GE, Flockhart IR, Smithen CE, Stratford IJ, Wardman P, Watts ME (1977) Electron-affinic sensitization VII: a correlation between structures, one-electron potentials and efficiencies of nitroimidazoles as hypoxic cell radiosensitizers. Radiat Res 67: 9–20 940937

[bib4] Airley R, Loncaster J, Davidson S, Bromley M, Roberts S, Patterson A, Hunter R, Stratford I, West C (2001) Glucose transporter glut-1 expression correlates with tumor hypoxia and predicts metastasis-free survival in advanced carcinoma of the cervix. Clin Cancer Res 7 (4): 928–934 11309343

[bib5] Armstrong AC, Dermime S, Allinson CG, Bhattacharyya T, Mulryan K, Gonzalez KR, Stern PL, Hawkins RE (2002) Immunization with a recombinant adenovirus encoding a lymphoma idiotype: induction of tumor-protective immunity and identification of an idiotype-specific T cell epitope. J Immunol 168: 3983–3991 11937555 10.4049/jimmunol.168.8.3983

[bib6] Atwell GJ, Rewcastle GW, Baguley BC, Denny WA (1989) Synthesis and antitumour activity of topologically related analogues of flavone acetic acid. Anticancer Drug Des 4: 161–169 2803464

[bib7] Bagshawe KD (1987) Antibody directed enzymes revive anti-cancer prodrugs concept. Br J Cancer 56: 531–532 3426915 10.1038/bjc.1987.237 PMC2001889

[bib8] Bagshawe KD (1995) Antibody-directed enzyme prodrug therapy for cancer: its theoretical basis and application. Mol Med Today 9: 424–431 10.1016/s1357-4310(95)90815-3 9415191

[bib9] Bagshawe KD, Sharma SK, Springer CJ, Antoniw P, Boden JA, Rogers GT, Burke PJ, Melton RG, Sherwood RF (1991) Antibody directed enzyme prodrug therapy (ADEPT): clinical report. Dis Markers 9: 233–238 1813213

[bib10] Bagshawe KD, Sharma SK, Springer CJ, Rogers GT (1994) Antibody directed enzyme prodrug therapy (ADEPT). A review of some theoretical, experimental and clinical aspects. Ann Oncol 5: 879–891 7696159 10.1093/oxfordjournals.annonc.a058725

[bib11] Bagshawe KD, Springer CJ, Searle F, Antoniw P, Sharma SK, Melton RG, Sherwood RF (1988) A cytotoxic agent can be generated selectively at cancer sites. Br J Cancer 58: 700–703 3265633 10.1038/bjc.1988.293 PMC2246864

[bib12] Bailey SM, Knox RJ, Hobbs SM, Jenkins TC, Mauger AB, Melton RG, Burke PJ, Connors TA, Hart IR (1996) Investigation of alternative prodrugs for use with *E.coli* nitroreductase in ‘suicide gene’ approaches to cancer therapy. Gene Therapy 12: 1143–1150 8986441

[bib13] Banerji U, O'Donnell A, Scurr M, Benson C, Brock C, Hanwell J, Stapleton S, Raynaud F, Simmons L, Turner A, Walton M, Workman P, Judson I (2002) A pharmacokinetically (PK)-pharmacodynamically (PD) driven Phase I trial of the HSP90 molecular chaperone inhibitor 17-allylamino 17-demethoxygeldanamycin (17AAG). Proc Am Assoc Cancer Res 43: 272 (abstr #1352)

[bib14] Bhatia J, Sharma SK, Chester KA, Pedley RB, Boden RW, Read DA, Boxer GM, Michael NP, Begent RHJ (2000) Catalytic activity of an *in vivo* tumor targeted anti-CEA scFv:carboxypeptidase G2 fusion protein. Int J Cancer 85: 571–577 10699932

[bib15] Begent RHJ, Verhaar MJ, Chester KA, Casey JL, Green AJ, Napier MP, Hope–Stone LD, Cushen N, Keep PA, Johnson CJ, Hawkins RE, Hilson AJW, Robson L (1996) Clinical evidence of efficient tumor targeting based on single–chain Fv antibody selected from a combinatorial library. Nat Med 2: 979–984 8782454 10.1038/nm0996-979

[bib16] Bibby MC (1991) Flavone acetic acid–an interesting novel therapeutic agent or just another disappointment? Br J Cancer 63: 3–5 1989662 10.1038/bjc.1991.2 PMC1971639

[bib17] Bibby MC, Double JA (1993) Flavone acetic acid–from laboratory to clinic and back. Anti-Cancer Drugs 4: 3–17 10.1097/00001813-199302000-00001 8457711

[bib18] Bibby MC, Double JA, Loadman PM, Duke CV (1989) Reduction of tumor blood flavone acetic acid: a possible component of therapy. J Natl Cancer Inst 81: 216–220 2911084 10.1093/jnci/81.3.216

[bib19] Bibby MC, Double JA, Phillips RM, Loadman PM (1987) Factors involved in the anti-cancer activity of the investigational agents LM985 (flavone acetic acid ester) and LM975 (flavone acetic acid). Br J Cancer 55: 159–163 3814485 10.1038/bjc.1987.32 PMC2002093

[bib20] Boehm MK, Corper AL, Wan T, Sohi MK, Sutton BJ, Thornton JD, Keep PA, Chester KA, Begent RH, Perkins SJ (2000) Crystal structure of the anti-(carcinoembryonic antigen) single-chain Fv antibody MFE-23 and a model for antigen binding based on intermolecular contacts. Biochem J 1 (346): 519–528 PMC1220881 10677374

[bib21] Boland MP, Knox RJ, Roberts JJ (1991) The differences in kinetics of rat and human DT diaphorase result in a differential sensitivity of derived cell lines to CB1954 (5-(aziridin-1-yl)-2,4-dinitrobenzamide). Biochem Pharmacol 41: 867–875 1901207 10.1016/0006-2952(91)90190-g

[bib22] Bolton MG, Kudekla A, Cassidy J, Calvert H (2002) Phase I studies of PG-paclitaxel(CT-2103) as a single agent and in combination with cisplatin. Proceedings of the 5th Int Symp Polymer Therap: Laboratory to Clinic, Cardiff, UK, p 19

[bib23] Bradshaw TD, Chua MS, Browne HL, Trapani V, Sausville EA, Stevens MF (2002) *In vitro* evaluation of amino acid prodrugs of novel antitumour 2-(4-amino-3-methylphenyl)benzothiazoles. Br J Cancer 86: 1348–1354 11953897 10.1038/sj.bjc.6600225 PMC2375326

[bib24] Bradshaw TD, Stevens MF, Westwell AD (2001) The discovery of the potent and selective antitumour agent 2-(4-amino-3-methylphenyl)benzothiazole (DF 203) and related compounds. Curr Med Chem 8 (2): 203–210 11172675 10.2174/0929867013373714

[bib25] Bridgewater JA, Springer CJ, Knox RJ, Minton NP, Michael NP, Collins MK (1995) Expression of the bacterial nitroreductase enzyme in mammalian cells renders them selectively sensitive to killing by the prodrug CB1954. Eur J Cancer 31A (13–14): 2362–2370 8652270 10.1016/0959-8049(95)00436-x

[bib26] Brocchini S, Duncan R (1999) Pendent drugs, release from polymers. In: Encyclopaedia of Controlled Drug Delivery, Mathiowitz E (ed) pp 786–816. New York: John wiley and Sons

[bib27] Burtles SS, Newell DR, Henrar REC, Connors TA (1995) Revisions of general guidelines for the preclinical toxicology of new cytotoxic anticancer agents in Europe. Eur J Cancer 31: 408–410 10.1016/0959-8049(94)00483-l 7786609

[bib28] Caiolfa VR, Zamal M, Fiorini A, Frigerio E, D'Argy R, Ghigleri A, Farao M, Angelucci F, Suarato A (2000) Polymer-bound camptothecin: initial biodistribution and antitumour activity studies. J Cont Rel 65: 105–120 10.1016/s0168-3659(99)00243-6 10699275

[bib29] Calvert AH, Harland SJ, Newell DR, Siddik ZH, Jones AC, McElwain TJ, Raju S, Wiltshaw E, Smith IE, Baker JM, Peckam MJ, Harrap KR (1982) Early clinical studies with *cis*-diammine-1,1-cyclobutane dicarboxylate platinum II. Cancer Chemother Pharmacol 9: 140–147 6761010 10.1007/BF00257742

[bib30] Calvert AH, Newell DR, Gumbrell LA, O'Reilly S, Burnell M, Boxall FE, Judson IR, Gore ME, Wiltshaw E (1989) Carboplatin dosage: prospective validation of a simple formula based on renal function. J Clin Oncol 7: 1748–1756 2681557 10.1200/JCO.1989.7.11.1748

[bib31] Ching LM, Baguley BC (1988) Enhancement of *in vitro* cytotoxicity of mouse peritoneal exudate cells by flavone acetic acid (NSC347512). Eur J Cancer Clin Oncol 24: 1521–1525 3181272 10.1016/0277-5379(88)90345-8

[bib32] Ching LM, Joseph WR, Crosier KE, Baguley BC (1994) Induction of tumour necrosis factor-alpha messenger RNA in human and murine cells by the flavone acetic acid analogue 5,6 dimethylxanthenone-4-acetic acid (NSC 640488). Cancer Res 54: 870–872 8313372

[bib33] Chua MS, Kashiyama E, Bradshaw TD, Stinson SF, Brantley E, Sausville EA, Stevens MF (2000) Role of CYP1A1 in modulation of antitumor properties of the novel agent 2-(4-amino-3-methylphenyl)benzothiazole (DF 203, NSC 674495) in human breast cancer cells. Cancer Res 60: 5196–5203 11016648

[bib34] Chung-Faye G, Palmer D, Anderson D, Clark J, Downes M, Baddeley J, Hussain S, Murray PI, Searle P, Seymour L, Harris PA, Ferry D, Kerr DJ (2001) Virus-directed, enzyme prodrug therapy with nitroimidazole reductase: a Phase I and pharmacokinetic study of its prodrug, CB1954. Clin Cancer Res 7 (9): 2662–2668 11555577

[bib35] Clark PA, Hostein I, Banerji U, Di Stefano F, Maloney A, Walton M, Judson I, Workman P (2000) Gene expression profiling of colon adenocarcinoma cells following inhibition of signal transduction by 17-allylamino-17-demethoxygeldanamycin, an inhibitor of the Hsp90 molecular chaperone. Oncogene 19: 4125–4133 10962573 10.1038/sj.onc.1203753

[bib36] Clive S, Webb DJ, MacLellan A, Young A, Byrne B, Robson L, Smyth JF, Jodrell DI (2001) Forearm blood flow and local responses to peptide vasodilators: a novel pharmacodynamic measure in the phase I trial of antagonist G, a neuropeptide growth factor antagonist. Clin Cancer Res 7: 3071–3078 11595697

[bib37] Cobb LM, Connors TA, Elson LA, Khan AH, Mitchley BC, Ross WC, Whisson ME (1969) 2,4-dinitro-5-ethyleneiminobenzamide (CB1954): a potent and selective inhibitor of the growth of the Walker carcinoma 256. Biochem Pharmacol 18: 1519–1527 4307990 10.1016/0006-2952(69)90267-6

[bib38] Connors TA, Farmer PB, Foster AB, Gilsenan AM, Jarman M, Tisdale MJ (1973) Metabolism of aniline mustard (*N*,*N*-di-(2-chloroethyl)aniline). Biochem Pharmacol 22: 1971–1980 4726563 10.1016/0006-2952(73)90079-8

[bib39] Connors TA, Jones M, Ross WCJ, Braddock PD, Khokhar AR, Tobe ML (1972) New platinum-complexes with anti-tumour activity. Chem Biol Interactions 5: 6415–6424 10.1016/0009-2797(72)90078-6 4652593

[bib40] Connors TA, Melzack DH (1971) Studies on the mechanism of action of 5-aziridinyl-2,4-dinitrobenzamide (CB1954), a selective inhibitor of the Walker tumour. Int J Cancer 7: 86–92 5546147 10.1002/ijc.2910070110

[bib41] Connors TA, Whisson ME (1965a) Drug-induced regression of large plasma cell tumours. Nature 205: 406 14243437 10.1038/205406a0

[bib42] Connors TA, Whisson ME (1965b) Cure of mice bearing advanced plasma cell tumours with aniline mustard. Nature 206: 689–691 5832853 10.1038/206689a0

[bib43] Connors TA, Whisson ME (1966) Cure of mice bearing advanced plasma cell tumours with aniline mustard: the relationship between glucuronidase activity and tumour sensitivity. Nature 210: 866–867 10.1038/210866b0 5958471

[bib44] Coombes RC, Haynes BP, Dowsett M, Quigley M, English J, Judson IR, Griggs LJ, Potter GA, McCague R, Jarman M (1995) Idoxifene: report of a Phase I study in patients with metastatic breast cancer. Cancer Res 55: 1070–1074 7866990

[bib45] Cummings J, MacLellan AJ, Mark M, Jodrell DI (1999) Development of a gradient elution high-performance liquid chromatography assay with ultraviolet detection for the determination in plasma of the anticancer peptide [Arg6, D-Trp7,9, mePhe8]-substance P (6-11) (antagonist G), its major metabolites and a C-terminal pyrene-labelled conjugate. J Chromatogr B Biomed Sci Appl 732 (2): 277–285 10517349 10.1016/s0378-4347(99)00294-7

[bib46] Dark GG, Hill SA, Prise VE, Tozer GM, Pettit GR, Chaplin DJ (1997) Combretastatin A4, an agent that displays potent and selective toxicity towards tumour vasculature. Cancer Res 57: 1829–1834 9157969

[bib47] Denekamp J (1982) Endothelial cell proliferation as a novel approach targeting tumour therapy. Br J Cancer 45: 136–139 7059456 10.1038/bjc.1982.16 PMC2010961

[bib48] Dhodapkar M, Rubin J, Reid JM, Burch PA, Pitot HC, Buckner JC, Ames MM, Suman VJ (1997) Phase I trial of temozolomide (NSC 362856) in patients with advanced cancer. Clin Cancer Res 7: 1093–1100 9815788

[bib49] Double JA (2002) Obituary of Tom Connors. Br J Cancer 8: 1205–1206

[bib50] Double JA, Bibby MC, Loadman PM (1986) Pharmacokinetics and anti-tumour activity of LM985 in mice bearing transplantable adenocarcinoma of the colon. Br J Cancer 54: 595–600 3778803 10.1038/bjc.1986.214 PMC2001488

[bib51] Double JA, Workman P (1977) A new high-glucuronidase tumour curable by aniline mustard therapy. Cancer Treatment Rep 61: 909 890696

[bib52] Dowsett M, MacNeill F, Mehta A, Newton C, Haynes B, Jones A, Jarman M, Lonning P, Powles TJ, Coombes RC (1991) Endocrine, pharmacokinetic and clinical studies of the aromatase inhibitor 3-ethyl-3-(4-pyridyl)piperidine-2,6-dione (‘pyridoglutethimide’) in postmenopausal breast cancer patients. Br J Cancer 64: 887–894 1931611 10.1038/bjc.1991.420 PMC1977449

[bib53] Duncan R (1992) Drug–polymer conjugates: potential for improved chemotherapy. Anticancer Drugs 3 (3): 175–210 1525399 10.1097/00001813-199206000-00001

[bib54] Duncan R, Kopecek J (1984) Soluble synthetic polymers as potential drug carriers. Adv Polymer Sci 57: 51–101

[bib55] Duncan R, Kopecek J, Rejmanova P, Lloyd JB (1983) Targeting of *N*-(2-hydroxypropyl) methacrylamide copolymers to liver by incorporation of galactose residues. Biochim Biophys Acta 755: 518–521 6824743 10.1016/0304-4165(83)90258-1

[bib56] Duncan R, Seymour LCW, Scarlett L, Lloyd JB, Rejmanova P, Kopecek J (1986) Fate of *N*-(2-hydroxypropyl)methacrylamide copolymers with pendant galactosamine residues after intravenous administration to rats. Biochim Biophys Acta 880: 62–71 3942780 10.1016/0304-4165(86)90120-0

[bib57] Duncan R, Spreafico F (1994) Polymer conjugates: pharmacokinetic considerations for design and development. Clin Pharmacokinet 27: 290–306 7834965 10.2165/00003088-199427040-00004

[bib58] EORTC/CRC Joint Steering Committee of the European Organisation for Research and Treatment of Cancer and the Cancer Research Campaign (1990) General guidelines for the preclinical toxicology of new cytotoxic anticancer agents in Europe. Eur J Cancer 26 (3): 411–414 2141502 10.1016/0277-5379(90)90244-n

[bib59] European Agency for the Evaluation of Medicinal Products (1999) Committee for Proprietary Medicinal Products. Note for guidance on the pre-clinical evaluation of anticancer medicinal products www.emea.eu.int - CPMP/SWP/997/96

[bib60] European Parliament and Council of the European Union (2001) Directive 2001/20/EC on conduct of clinical trials. Official J of the Eur Communities L121: 34–44

[bib61] Fitzsimmons SA, Workman P, Grever M, Paul K, Camelier R, Lewis AD (1996) Reductase expression across the National Cancer Institute tumor cell line panel: correlation with sensitivity to mitomycin C and EO9. J Natl Cancer Inst 88: 259 8614004 10.1093/jnci/88.5.259

[bib62] Galbraith SM, Rustin GJ, Lodge MA, Taylor NJ, Stirling JJ, Jameson M, Thompson P, Hough D, Gumbrell L, Padhani AR (2002) Effects of 5,6-dimethylxanthenone-4-acetic acid on human tumor microcirculation assessed by dynamic contrast-enhanced magnetic resonance imaging. J Clin Oncol 20: 3826–3840 12228202 10.1200/JCO.2002.09.144

[bib63] Galbraith SM, Taylor NJ, Lodge M, Tozer GM, Baddeley H, Wilson I, Prise VE, Rustin GJS (2000) Combretastatin A4 phosphate targets vasculature in animal and human tumours. Br J Cancer 83 (1): 12

[bib64] Gianasi E, Wasil M, Evagorou EG, Keddle A, Wilson G, Duncan R (1999) HPMA copolymer platinates as novel antitumour agents: *in vitro* properties, pharmacokinetics and antitumour activity. Eur J Cancer 3: 994–1002 10.1016/s0959-8049(99)00030-1 10533484

[bib65] Green NK, Youngs DJ, Neoptolemos JP, Friedlos F, Knox RJ, Springer CJ, Anlezark GM, Michael NP, Melton RG, Ford MJ, Young LS, Kerr DJ, Searle PF (1997) Sensitization of colorectal and pancreatic cancer cell lines to the prodrug 5-(aziridin-1-yl)-2,4-dinitrobenzamide (CB1954) by retroviral transduction and expression of the E. coli nitroreductase gene. Cancer Gene Ther 4 (4): 229–238 9253508

[bib66] Grosios K, Holwell SE, McGown AT, Pettit GR, Bibby MC (1999) *In vivo* and *in vitro* evaluation of combretastatin A4 and its sodium phosphate prodrug. Br J Cancer 81: 1318–1327 10604728 10.1038/sj.bjc.6692174 PMC2362967

[bib67] Hawkins RE, Russell SJ, Stevenson FK, Hamblin TJ (1997) A pilot study of idiotypic vaccination for follicular B-cell lymphoma using a genetic approach. Human Gene Ther 8: 1287–1299 9215745 10.1089/hum.1997.8.10-1287

[bib68] Haynes BP, Jarman M, Dowsett M, Mehta A, Lonning P, Griggs LJ, Jones A, Powles T, Stein R, Coombes RC (1991) Pharmacokinetics and pharmacodynamics of the aromatase inhibitor 3-ethyl-3-(4-pyridyl)piperidine-2,6-dione in patients with postmenopausal breast cancer. Cancer Chemother Pharmacol 27: 367–372 1847846 10.1007/BF00688859

[bib69] Hendriks HR, Pizao PE, Berger DP, Kooistra K, Bibby MC, Boven E, Dreef-van der Meulen HC, Henrar REC, Fiebig HH, Double JA, Hornstra HW, Pinedo HM, Workman P, Schwartzsmann G (1993) EO9: a novel bioreductive alkylating indoloquinone with preferential solid tumour activity and lack of bone marrow toxicity in preclinical models. Eur J Cancer 29A: 897 8484984 10.1016/s0959-8049(05)80434-4

[bib70] Hill S, Williams KB, Denekamp, J (1989) Vascular collapse after flavone acetic acid: a possible mechanism of its anti-tumour action. Eur J Cancer Clin Oncol 25: 1419–1424 2591434 10.1016/0277-5379(89)90099-0

[bib71] Hollingshead MG, Alley MC, Camalier RF, Abbot BJ, Mayo JG, Malspeis L, Grever MR (1995) *In vivo* cultivation of tumour cells in hollow fibres. Life Sci 57: 131–141 7603295 10.1016/0024-3205(95)00254-4

[bib72] Horwich A, Holliday SB, Deacon JM, Peckham MJ (1986) A toxicity and pharmacokinetic study in man of the hypoxic-cell radiosensitizer RSU-1069. Br J Radiol 59: 1238–1240 3542110 10.1259/0007-1285-59-708-1238

[bib73] Hostein I, Robertson D, Di Stefano F, Workman P, Clarke PA (2001) Inhibition of signal transduction by the Hsp90 17-allylamino-17-demethoxygeldanamycin results in cytostasis and apoptosis. Cancer Res 61: 4003–4009 11358818

[bib74] Hughes AN, Griffin MJ, Newell DR, Calvert AH, Johnston A, Kerr B, Lee C, Liang B, Boddy AV (2000) Clinical pharmacokinetic and *in vitro* combination studies of nolatrexed dihydrochloride (AG337, Thymitaq (TM)) and paclitaxel. Br J Cancer 82: 1519–1527 10789718 10.1054/bjoc.2000.1172 PMC2363406

[bib75] Jodrell DI (1999) Formula-based dosing for carboplatin. Eur J Cancer 35: 1299–1301 10658517 10.1016/s0959-8049(99)00127-6

[bib76] Jodrell DI, Bowman A, Rye R, Byrne B, Boddy A, Rafi I, Taylor GA, Johnston A, Clendeninn NJ (1999) A phase I study of the lipophilic thymidylate synthase inhibitor Thymitaq (nolatrexed dihydrochloride) given by 10-day oral administration. Br J Cancer 79: 915–920 10070890 10.1038/sj.bjc.6690146 PMC2362691

[bib77] Kelland LR, Sharp SY, Rogers PM, Myers TG, Workman P (1999) DT-diaphorase expression and tumor cell sensitivity to 17-allylamino, 17-demethoxygeldanamycin, an inhibitor of heat shock protein 90. J Natl Cancer Inst 91: 1940–1949 10564678 10.1093/jnci/91.22.1940

[bib78] Kerr DJ, Kaye SB, Cassidy J, Dutta S, Setanoians A, Forrest G, Cunningham D, Soukop M, Vezin WR (1985) A clinical pharmacokinetic study of LM985 and LM975. Br J Cancer 52: 467

[bib79] Kerr DJ, Kaye SB, Cassidy J, Bradley C, Rankin EM, Adams L, Setanoians A, Young T, Forrest G, Soukop M, Clacel C (1987) Phase I and pharmacokinetic study of flavone acetic acid. Cancer Res 47: 6776–6781 3677106

[bib80] Kerr DJ, Maughan T, Newlands E, Rustin G, Bleehan NM, Lewis C, Kaye SB (1989) Phase II trials of flavone acetic acid in advanced malignant melanoma and colorectal carcinoma. Br J Cancer 60: 104–106 2803908 10.1038/bjc.1989.230 PMC2247346

[bib81] Knox RJ, Boland MP, Friedlos F, Coles B, Southan C, Roberts JJ (1988a) The nitroreductase enzyme in Walker cells that activates 5-(aziridin-1-yl)-2,4-dinitrobenzamide (CB1954) to 5-(aziridin-1-yl)-4-hydroxylamino-2-nitrobenzamide is a form of NAD(P)H dehydrogenase (quinone) (EC 1.6.99.2). Biochem Pharmacol 37: 4671–4677 3144286 10.1016/0006-2952(88)90336-x

[bib82] Knox RJ, Friedlos F, Jarman M, Roberts JJ (1988b) A new cytotoxic, DNA interstrand crosslinking agent, 5-(aziridin-1-yl)-4-hydroxylamino-2-nitrobenzamide, is formed from 5-(aziridin-1-yl)-2,4-dinitrobenzamide (CB1954) by a nitroreductase enzyme in Walker carcinoma cells. Biochem Pharmacol 37: 4661–4669 3202902 10.1016/0006-2952(88)90335-8

[bib83] Knox RJ, Friedlos F, Marchbank T, Roberts JJ (1991) Bioactivation of CB1954: reaction of the active 4-hydroxylamino derivative with thioesters to form the ultimate DNA–DNA interstrand crosslinking species. Biochem Pharmacol 42: 1691–1697 1930294 10.1016/0006-2952(91)90503-w

[bib84] Knox RJ, Friedlos F, Sherwood RF, Melton RG, Anlezark GM. (1992) The bioactivation of 5-(aziridin-1-yl)-2,4-dinitrobenzamide (CB1954)–II. A comparison of an Escherichia coli nitroreductase and Walker DT diaphorase. Biochem Pharmacol 44: 2297–2301 1472095 10.1016/0006-2952(92)90672-6

[bib85] Knox RJ, Jenkins TC, Hobbs SM, Chen S, Melton RG, Burke PJ (2000a) Bioactivation of 5-(aziridin-1-yl)-2,4-dinitrobenzamide (CB1954) by human NAD(P)H quinone oxidoreductase 2: a novel co-substrate-mediated antitumor prodrug therapy. Cancer Res 60: 4179–4186 10945627

[bib86] Knox J, Melton RG, Sharma SK, Burke PJ (2000b) Activation of CB1954 by NQO2–a novel and direct anti-tumour therapy. Br J Cancer 83 (1): 70

[bib87] Kreitman RJ, Wilson WH, Bergeron K, Raggio M, Stetler-Stevenson M, FitzGerald DJ, Pastan I (2001) Efficacy of the anti-CD22 recombinant immunotoxin BL22 in chemotherapy-resistant hairy-cell leukemia. N Engl J Med 345 (4): 241–247 11474661 10.1056/NEJM200107263450402

[bib88] Laws AL, Matthew AM, Double JA, Bibby MC (1995) Preclinical *in vitro* and *in vivo* activity of 5,6-dimethylxanthenone-4-acetic acid. Br J Cancer 71: 1204–1209 7779712 10.1038/bjc.1995.234 PMC2033820

[bib89] Loaiza-Perez AI, Trapani V, Hose C, Singh SS, Trepel JB, Stevens MF, Bradshaw TD, Sausville EA (2002) Aryl hydrocarbon receptor mediates sensitivity of MCF-7 breast cancer cells to antitumor agent 2-(4-amino-3-methylphenyl) benzothiazole. Mol Pharmacol 61 (1): 13–19 11752201 10.1124/mol.61.1.13

[bib90] Mann J, Haase-Held M, Springer CJ, Bagshawe KD (1990) Synthesis of an *N*-mustard prodrug. Tetrahedron 46: 5377–5382

[bib91] Matsumura Y, Maeda H (1986) A new concept for macromolecular therapies in cancer chemotherapy: mechanism of tumouritropic accumulation of proteins and the antitumour agent SMANCS. Cancer Res 6: 6387–6392 2946403

[bib92] McGown AT, Fox BW (1989) Structural and biochemical comparison of the anti-mitotic agents colchicine, combretastatin A4 and amphethinile. Anti-Cancer Drug Des 3: 249–254 2930627

[bib93] McNally V, Patterson AV, Williams K, Cowen RL, Stratford IJ, Jaffar M (2002) Antiangiogenic, bioreductive and gene therapy approaches to the treatment of hypoxic tumours. Current Pharm Des 8: 1319–1333 10.2174/1381612023394548 12052210

[bib94] McNeish IA, Green NK, Gilligan MG, Ford MJ, Mautner V, Young LS, Kerr DJ, Searle PF (1998) Virus directed enzyme prodrug therapy for ovarian and pancreatic cancer using retrovirally delivered E. coli nitroreductase and CB1954. Gene Therapy 5 (8): 1061–1069 10326029 10.1038/sj.gt.3300744

[bib95] McVie JG, ten Bokkel Huinink WW, Simonetti G, Dubbelman R (1984) Phase I trial of *N*-methylformamide. Cancer Treat Rep 68 (4): 607–610 6325001

[bib96] Meerum Terwogt JM, ten Bokkel Huinink WW, Schellens JH, Schot M, Mandjes IA, Zurlo MG, Rocchetti M, Rosing H, Koopman FJ, Beijnen JH (2001) Phase I clinical and pharmacokinetic study of PNU166945, a novel water-soluble polymer-conjugated prodrug of paclitaxel. Anticancer Drugs 12: 315–323 11335787 10.1097/00001813-200104000-00003

[bib97] Mehta LK, Hobbs S, Chen S, Knox RJ, Parrick J (1999) Phthalimide analogues of CB1954: synthesis and bioactivation. Anticancer Drugs 10: 777–783 10573210 10.1097/00001813-199909000-00011

[bib98] Melton RG, Searle F, Sherwood RF, Bagshawe KD, Boden JA (1990) The potential of carboxypeptidase G2: antibody conjugates as anti-tumour agents. II. *In vivo* localising and clearance properties in a choriocarcinoma model. Br J Cancer 61: 420–424 2328209 10.1038/bjc.1990.92 PMC1971295

[bib99] Monks A, Scuderio DA, Johnson GS, Paull KD, Sausville EA (1997) The NCI anti-cancer drug screen: a smart screen to identify effectors of novel targets. Anticancer Drug Des 12: 533–541 9365500

[bib100] Napier MP, Sharma SK, Springer CJ, Bagshawe KD, Green AJ, Martin J, Stribbling SM, Cushen N, O'Malley D, Begent RHJ (2000) Antibody-directed enzyme prodrug therapy: efficacy and mechanism of action in colorectal carcinoma. Clin Cancer Res 6: 765–772 10741695

[bib101] Newell H (2002) Be brilliant!–a tribute to Tom Connors. Eur J Cancer 38: 1163–1164

[bib102] Newell DR, Burtles SS, Fox BW, Jodrell DI, Connors TA (1999) Evaluation of rodent-only toxicology for early clinical trials with novel cancer therapeutics. Br J Cancer 81: 760–768 10555743 10.1038/sj.bjc.6690761 PMC2374299

[bib103] Newlands ES, Blackledge G, Slack JA, Goddard C, Brindley CJ, Holden L, Stevens MFG (1985) Phase I clinical trial of mitozolomide. Cancer Treat Rep 69: 801–805 4016790

[bib104] Newlands ES, Blackledge GRP, Slack JA, Rustin GJS, Smith DB, Stuart NSA, Quarterman CP, Hoffman R, Stevens MFG, Brampton MH, Gibson AC (1992) Phase I trial of temozolomide (CCRG 81045: M and B 39831: NSC 362856). Br J Cancer 65: 287–291 1739631 10.1038/bjc.1992.57 PMC1977719

[bib105] Newlands ES, Stevens MF, Wedge SR, Wheelhouse RT, Brock C (1997) Temozolomide: a review of its discovery, chemical properties, pre-clinical development and clinical trials. Cancer Treat Rev 23: 35–61 9189180 10.1016/s0305-7372(97)90019-0

[bib106] Patterson LH, McKeown SR (2000) AQ4N: a new approach to hypoxia-activated cancer chemotherapy. Br J Cancer 83: 12 1589–1593. 10.1054/bjoc.2000.1564 PMC2363465 11104551

[bib107] Pettit GR, Singh SB, Hamel E, Lin CM, Alberts DS, Garia-Kendall D (1989) Isolation and structure of the strong cell growth and tubulin inhibitor combretastatin A4. Experientia 45: 209–211 2920809 10.1007/BF01954881

[bib108] Pettit GR, Singh SB, Niven Ml, Hamel E, Schmidt JM (1987) Isolation, structure and synthesis of combretastatins A1 and B1, potent new inhibitors of microtubule assembly, derived from *Combretum caffrum*. J Nat Prod 50: 119–131 3598594 10.1021/np50049a016

[bib109] Pettit GR, Temple C, Narayanan Vl, Varma R, Simpson MJ, Boyd MR, Rener GA, Bansal N (1995) Antineoplastic agents 322. Synthesis of combretastatin A4 prodrugs. Anti-Cancer Drug Des 10: 299–309 7786396

[bib110] Plumb JA, Workman P (1994) Unusually marked hypoxic sensitisation to indoloquinone EO9 and mitomycin C in a human colon-tumour cell line that lacks DT-diaphorase activity. Int J Cancer 56: 134 8262670 10.1002/ijc.2910560124

[bib111] Potter GA, Barrie SE, Jarman M, Rowlands MG (1995) Novel steroidal inhibitors of human cytochrome *P*450_17_*_α_* (17*α*-hydroxylase-C_17,20_-lyase): potential agents for the treatment of prostatic cancer. J Med Chem 38: 2463–2471 7608911 10.1021/jm00013a022

[bib112] Rafi I, Boddy AV, Calvete JA, Taylor GA, Newell DR, Bailey NP, Lind MJ, Green M, Hine J, Johnston A, Clendeninn N, Calvert AH (1998) Preclinical and Phase I clinical studies with the non-classical antifolate thymidylate synthase inhibitor nolatrexed dihydrochloride given by prolonged administration in patients with solid tumours. J Clin Oncol 16: 1131–1141 9508200 10.1200/JCO.1998.16.3.1131

[bib113] Rafi I, Taylor GA, Calvete JA, Boddy AV, Balmanno K, Bailey N, Lind M, Calvert AH, Webber S, Jackson RJ, Johnston A, Clendeninn N, Newell DR (1995) Clinical pharmacokinetic and pharmacodynamic studies with the nonclassical antifolate thymidylate synthase inhibitor 3,4-dihydro-2-amino-6-methyl-4-oxo-5-(4-pyridylthio)-quinazoline dihydrochloride (AG337) given by 24-hour intravenous infusion. Clin Cancer Res 1: 1275–1284 9815922

[bib114] Rewcastle GW, Atwell GJ, Baguley BC, Boyd M, Thomsen LL, Zhuang L, Denny WA (1991c) Potential antitumour agents. 63. Structure–activity relationships for side-chain analogues of the colon 38 active agent 9-oxo-9H-xanthene-4-acetic acid. J Med Chem 34: 2864–2870 1895304 10.1021/jm00113a027

[bib115] Rewcastle GW, Atwell GJ, Baguley BC, Calveley SB, Denny WA (1989) Potential anti-tumour agents. 58. Synthesis and structure–activity relationships of substituted xanthenone-4-acetic acid against the colon 38 tumour *in vivo*. J Med Chem 32: 793–799 2704025 10.1021/jm00124a012

[bib116] Rewcastle GW, Atwell GJ, Palmer BD, Boyd PDW, Baguley BC, Denny WA (1991b) Potential antitumour agents. 62. Structure–activity relationships for tricyclic compounds related to the colon tumour active drug 9-oxo-9*H*-xanthene-4-acetic acid. J Med Chem 34: 491–496 1995870 10.1021/jm00106a003

[bib117] Rewcastle GW, Atwell GJ, Zhuang L, Baguley BC, Denny WA (1991a) Potential anti-tumour agents. 61. Structure–activity relationships for in vivo colon 38 activity among di-substituted 9-oxo-9H-xanthene-4-acetic acids. J Med Chem 34: 217–222 1992120 10.1021/jm00105a034

[bib118] Riley R, Workman P (1992) DT-diaphorase in cancer chemotherapy. Biochem Pharmacol 43: 1657 1575764 10.1016/0006-2952(92)90694-e

[bib119] Ringsdorf H (1975) Structure and properties of pharmacologically active polymers. J Pharm Sci Polym Symp 51: 135–153

[bib120] Roberts JJ, Friedlos F, Knox RJ (1986) CB1954 (2,4-dinitro-5-aziridinyl benzamide) becomes a DNA interstrand crosslinking agent in Walker tumour cells. Biochem Biophys Res Commun 140: 1073–1078 3778483 10.1016/0006-291x(86)90744-8

[bib121] Sabbatini P, Aghajanian C, Hensley M, Pezzulli S, Oflaherty C, Soigner S, Lovegren M, Esch J, Funt S, Odujinrin O, Warner M, Bolton MG, Spriggs D (2002) Early findings in a Phase I/II study of PG-paclitaxel (CT-2103) in recurrent ovarian or primary peritoneal cancer. Proceedings of the 5th Int Symp Polymer Therap: Laboratory to Clinic, Cardiff, UK, p 20

[bib122] Seddon BS, Maxwell RJ, Honess DJ, Grimshaw R, Raynaud F, Tozer G, Workman P (2002a) Validation of the fluorinated 2-nitroimidazole SR 4554 as a hypoxia marker for non-invasive magnetic resonance spectroscopy: studies using a tumour model with oxygenation characteristics similar to many human tumours. Clin Cancer Res 8: 2323–2335 12114437

[bib123] Seddon BM, Payne GS, Simmons LM, Grimshaw R, Tan S, Raynaud F, Leach MO, Judson MI, Workman P (2002b) Phase I pharmacokinetics and magnetic resonance spectroscopic study of the non-invasive hypoxia probe SR-4554. Proc Am Soc Clin Oncol 2002 (21): 91b (abstract 2176)

[bib124] Seymour LW, Ferry DR, Anderson D, Hesslewood S, Julyan PJ, Payner R, Doran J, Young AM, Burtles S, Kerr DJ (2002) Hepatic drug targeting: Phase I evaluation of polymer bound doxorubicin. J Clin Oncol 20: 1668–1676 11896118 10.1200/JCO.2002.20.6.1668

[bib147] Sharma SK, Boden JA, Springer CJ, Burke PJ and Bagshaw KD (1994) Antibody-directed enzyme prodrug therapy (ADEPT). A three-phase study in ovarian tumour xenografts. Cell Biophys 24/25: 219–228 10.1007/BF02789232 7736526

[bib125] Sharp SY, Kelland LR, Valenti MR, Brunton LA, Hobbs S, Workman P (2000) Establishment of an isogenic human colon tumor model for NQO1 gene expression: application to investigate the role of DT-diaphorase in bioreductive drug activation *in vitro* and *in vivo*. Mol Pharmacol 58: 1146–1155 11040064 10.1124/mol.58.5.1146

[bib126] Siim BG, Lee AE, Shalal-Zwain S, Pruijn FB, McKeage MJ, Wilson WR (2003) Marked potentiation of the antitumour activity of chemotherapeutic drugs by the antivascular agent 5,6-dimethylxanthenone-4-acetic acid (DMXAA). Cancer Chemother Pharmacol 51: 43–52 12497205 10.1007/s00280-002-0529-0

[bib127] Spencer DR, Robson L, Purdy D, Whitelegg NR, Michael NP, Bhatia J, Sharma S, Rees AR, Minton NP, Begent RHJ, Chester KA (2002) A strategy for mapping and neutralising conformational epitopes on protein therapeutics. Proteomics 2: 271–279 11921443 10.1002/1615-9861(200203)2:3<271::aid-prot271>3.0.co;2-w

[bib128] Springer CJ, Antoniw P, Bagshawe KD, Searle F, Bisset GM, Jarman M (1990) Novel prodrugs which are activated to cytotoxic alkylating agents by carboxypeptidase G2. J Med Chem 33: 677–681 2299634 10.1021/jm00164a034

[bib129] Springer CJ, Bagshawe KD, Sharma SK, Searle F, Boden JA, Antoniw P, Burke PJ, Rogers GT, Sherwood RF, Melton RG (1991) Ablation of human choriocarcinoma xenografts in nude mice by antibody-directed enzyme prodrug therapy (ADEPT) with three novel compounds. Eur J Cancer 27: 1361–1366 1835849 10.1016/0277-5379(91)90010-b

[bib130] Stevens MF, Newlands ES (1993) From triazines and triazenes to temozolomide. Eur J Cancer 29A (7): 1045–1047 8499135 10.1016/s0959-8049(05)80221-7

[bib131] Stevenson JP, Gallagher M, Sun W, Algazy K, Vaughn DJ, Haller DG, Hiller K, Halloran L, O'Dwyer J (2000) Phase I/pharmacokinetic trial of the endothelial toxin combretastatin A4P (CA4P) administered as an IV bolus on a daily ×5 schedule every 21 days. Proc Am Assoc Cancer Res 41: 54

[bib132] Stratford IJ, Workman P (1998) Bioreductive drugs into the next millennium. Anti-Cancer Drug Des 13: 519–528 9755716

[bib133] Stuart NSA, Crawford SM, Blackledge GRP, Newlands ES, Slack J, Hoffman R, Stevens MFG (1989) A Phase I study of meta-azidopyrimethamine ethanesulphonate (MZPES)–a new dihydrofolate reductase inhibitor. Cancer Chemother Pharmacol 23: 308–310 2706736 10.1007/BF00292409

[bib134] Suggit M, Swaine DJ, Pettit GR, Bibby MC (2002) Characterisation of the hollow fibre assay for the determination of tubulin interaction *in vivo*. Eur J Cancer 38 (Suppl 7): S39

[bib135] Timmerman JM, Czerwinski DK, Davis TA, Hsu FJ, Benike C, Hao ZM, Taidi B, Rajapaksa R, Caspar CB, Okada CY, van Beckhoven A, Liles TM, Engleman EG, Levy R (2002) Idiotype-pulsed dendritic cell vaccination for B-cell lymphoma: clinical and immune responses in 35 patients. Blood 99: 1517–1526 11861263 10.1182/blood.v99.5.1517

[bib136] Vasey P, Twelves C, Kaye S, Wilson P, Morrison R, Duncan R, Thomson A, Hilditch T, Murray T, Burtles S, Cassidy J (1999) Phase I clinical and pharmacokinetic study of PKI (HPMA copolymer doxorubicin) first member of a new class of chemotherapeutics agents: drug–polymer conjugates. Clin Cancer Res 5: 83–94 9918206

[bib137] Weedon SJ, Green NK, McNeish IA, Gilligan MG, Mautner V, Wrighton CJ, Mountain A, Young LS, Kerr DJ, Searle PF (2000) Sensitisation of human carcinoma cells to the prodrug CB1954 by adenovirus vector-mediated expression of E. coli nitroreductase. Int J Cancer 86 (6): 848–854 10842200 10.1002/(sici)1097-0215(20000615)86:6<848::aid-ijc14>3.0.co;2-b

[bib138] Workman P (1978) Inhibition of human prostatic tumour acid phosphatases by *N,N*-*p*-di-2-chloroethylaminophenol,*N*,*N*-*p*-di-2-chloroethylamino-phenyl phosphate and other disfunctional nitrogen mustards. Chem-Biol Interactions 20: 103 10.1016/0009-2797(78)90085-6 630640

[bib139] Workman P (1994) Enzyme-directed bioreductive drug development revisited: a commentary on recent progress and future prospects with emphasis on quinone anticancer agents and quinone metabolizing enzymes, particularly DT-diaphorase. Oncol Res 6: 461 7620214

[bib140] Workman P, Binger M, Kooistra KL (1992) Pharmacokinetics, distribution and metabolism of the novel bioreductive alkylating indoloquinone EO9 in rodents. Int J Radiat Oncol Biol Phys 22: 713–716 1544843 10.1016/0360-3016(92)90509-g

[bib141] Workman P, Double JA (1978) Enzyme-activated antitumor agents–IV. Comparative kinetics of *N,N-p*-di-2-chloroethylaminophenyl phosphate hydrolysis catalysed by phosphatases of normal and neoplastic tissues. Biochem Pharmacol 27: 199 623675 10.1016/0006-2952(78)90301-5

[bib142] Workman P, Kaye SB (Eds) (2002) Translating basic cancer research into new cancer therapies. Trends Guide to Cancer Therapeutics, Supplement to Trends in Molecular Medicine. Amsterdam: Elsevier 10.1016/s1471-4914(02)02319-5 11927279

[bib143] Workman P, Morgan JE, Talbot K, Wright KA, Donaldson J, Twentyman PR (1986b) CB 1954 revisited. II. Toxicity and antitumour activity. Cancer Chemother Pharmacol 16 (1): 9–14 3940225 10.1007/BF00255279

[bib144] Workman P, White RA, Talbot K (1986a) CB 1954 revisited. I. Disposition kinetics and metabolism. Cancer Chemother Pharmacol 16 (1): 1–8 3940216 10.1007/BF00255278

[bib145] Wu K, Knox R, Sun XZ, Joseph P, Jaiswal AK, Zhang D, Deng PS, Chen S (1997) Protein catalytic properties of NAD(P)H:quinone oxidoreductase-2 (NQO2), a dihydronicotinamide riboside dependent oxidoreductase. Arch Biochem Biophys 347 (2): 221–228 9367528 10.1006/abbi.1997.0344

[bib146] Zwi LJ, Baguley BC, Gavin JB, Wilson WR (1989) Blood flow failure as a major determinant in the anti-tumour action of flavone acetic acid. J Natl Cancer Inst 81: 1005–1013 2733044 10.1093/jnci/81.13.1005

